# Anion-specific fluorescence enhancement *via* counterion modulation in biphenyl-based porous cage salts

**DOI:** 10.1039/d6sc06722k

**Published:** 2026-10-22

**Authors:** Rachel M. Sabatino, Merissa N. Morey, Alex Lovstedt, Eric D. Bloch

**Affiliations:** a Department of Chemistry, Indiana University Bloomington IN 47405 USA edbloch@iu.edu

## Abstract

Porous coordination cages offer a versatile platform for integrating photophysical functionality into well-defined molecular architectures; however, many widely studied cages are intrinsically non-emissive. Here, we report counterion-dependent fluorescence switching in biphenyl-functionalized zirconium porous cages, in which anion identity strongly influences emission behavior. The cage exhibits bright fluorescence when isolated as a chloride salt, while the corresponding triflate salt is essentially non-emissive despite the same underlying Zr–bpdc connectivity. Systematic counterion exchange and qualitative salt screening show that fluorescence enhancement from the triflate form depends chiefly on anion identity rather than the accompanying cation, with nitrate giving the largest enhancement. These results identify counterion modulation as a simple strategy for tuning fluorescence in zirconium porous cages and highlight the importance of anion identity in shaping their solid-state photophysical behavior.

## Abstract 2

Exchanging triflate for alternative anions enhances fluorescence in porous biphenyl-based zirconium cages.

## Graphical abstract

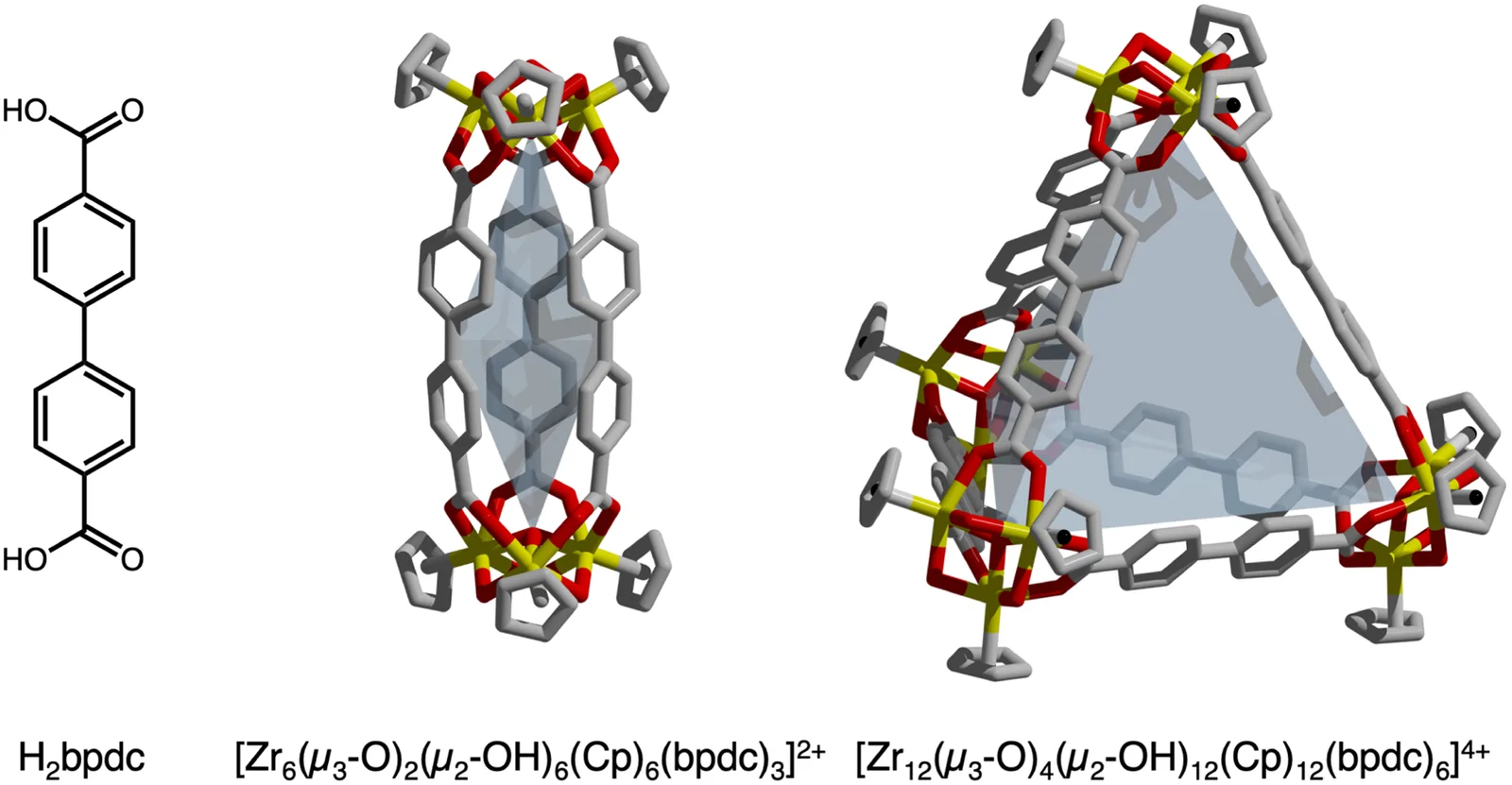

## Introduction

The optical properties of advanced porous materials have been widely leveraged for diverse applications ranging from catalysis^1^ and pollution remediation^2^ to quantum information science,^3^ chemical sensing,^4^ and photonic energy management.^5^ Hybrid porous materials, most prominently metal–organic frameworks (MOFs) and porous coordination cages (PCCs)/metal–organic polyhedra (MOPs), offer a distinct advantage in this space because both their inorganic nodes and organic linkers can be systematically modified to tune photophysical behavior.^6–8^ Beyond primary synthesis, the solid-state composition of these materials is also highly adaptable, as post-synthetic modification,^9^ counterion exchange,^10^ or the reversible encapsulation of guests can all alter the electronic environment of embedded chromophores.^11^ Such guest- or ion-induced optical changes have been used as the basis for selective sensing,^12^ where variations in emission intensity,^13^ wavelength,^14^ or lifetime report on the presence of specific analytes.^15^ Examples include nitroaromatic quenching in organic cages,^16^ lanthanide-based cages that enhance or suppress NIR emission upon anion binding,^17^ and organic fluorophore-loaded cages that respond to metal ions or reactive oxygen species.^18^

While a subset of metal–organic cages exhibit intrinsic fluorescence arising from metal-centered chromophores or specific ligands,^19,20^ including zirconium-based cages used for nitrite or phosphate anion sensing,^21,22^ many widely studied cage architectures, including calixarene-capped coordination cages,^23^ paddlewheel-based M_24_L_24_ assemblies,^24^ and cyclopentadienyl-capped zirconium cages, are essentially non-emissive by nature.^25^ In these systems, the metal nodes and their bridging carboxylates provide structural rigidity required for porosity but do not contribute appreciably to photoluminescence, and in many cases can even promote nonradiative decay pathways.^26^ As a result, introducing fluorescence into such cages requires judicious ligand design,^27^ typically by incorporating π-conjugated linkers or donor–acceptor motifs whose photophysical properties are preserved or even enhanced by the cage structure.^28,29^

In a simple yet powerful iteration of this strategy, biphenyl dicarboxylic acid offers an inherently fluorescent scaffold that can be incorporated directly into the structure of Zr-based porous solids.^29,30^ The biphenyl chromophore displays characteristic absorption near 280 nm, with emission centered at 345 nm in methanol, providing a built-in optical reporter whose behavior can potentially be modulated by the surrounding solid-state environment,^31^ guest molecules,^32^ or counterions.^33^ The rigid framework of porous cages restricts free rotation or vibration of the chromophore, thus blocking nonradiative decay pathways and enhancing fluorescence of the whole solid.^34^ This approach transforms otherwise non-emissive porous cages into photofunctional materials whose emission can serve as a sensitive probe of local chemical and structural changes.^35^

During ongoing efforts in our laboratory to prepare porous salts incorporating fluorescent anions,^36^ we made an unexpected observation: the biphenyl-based Zr cage itself exhibited bright solid-state fluorescence when isolated as a simple chloride salt, despite the absence of any intentionally introduced emissive components. Previously published work examined the solid-state photoluminescence properties of a biphenyl-based Zr cage, stating that the cage's emission at 404 nm (*λ*_ex_ = 330) stems from the biphenyl linker.^29^ In contrast, the corresponding triflate salt was essentially non-emissive under identical conditions. This serendipitous fluorescence contrast, first noticed during routine anion-exchange experiments, suggested that the optical properties of the cage were highly sensitive to its counterion environment. Importantly, neither chloride nor triflate ions possess inherent fluorescence, indicating that the emission originates from the biphenyl linker and is modulated by the local structural or electronic effects imposed by the anion.

This unexpected counterion dependence prompted us to investigate how anion identity influences cage fluorescence. Because these cage salts can undergo anion metathesis, we envisioned that the biphenyl-functionalized cage might provide a simple platform for examining how different anions restore or suppress fluorescence through their influence on packing, ion pairing, or excited-state decay pathways. The present study examines a family of chloride-, triflate-, and nitrate-containing cage compositions and shows that the emission behavior is strongly counterion-dependent, with distinct ion-specific responses in the solid state.

## Results and discussion

Our initial work with zirconium-based cages focused on understanding how cage geometry and structure vary as a function of ligand identity.^37^ For linear dicarboxylate linkers, these systems can adopt at least two distinct structure types featuring trinuclear zirconium nodes bridged by dicarboxylate ligands (Fig. 1). The resulting architectures are either a V_4_L_6_ tetrahedron or a V_2_L_3_ cigar (lantern) motif, with the favored structure depending on reaction conditions, starting materials, and anion identity. It is generally accepted that conversion of the V_4_L_6_ tetrahedron to two equivalents of the V_2_L_3_ cigar is thermodynamically favored and irreversible.^38^ In the course of investigating this transformation, we observed that the inherent fluorescence of the cage is highly dependent on the associated anion (Fig. 2).

**Fig. 1:**
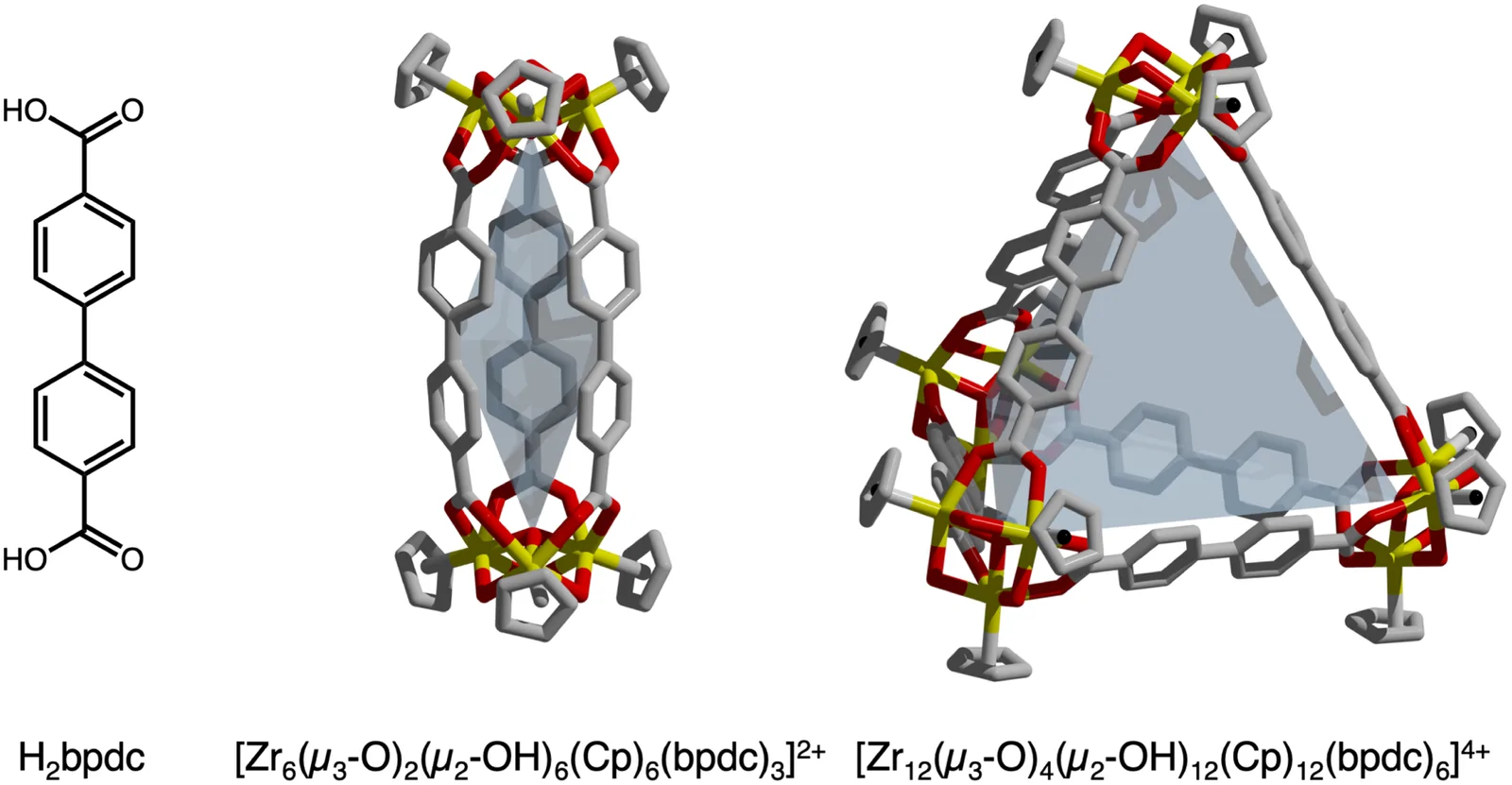
Structures of 4,4′-biphenyldicarboxylic acid (H_2_bpdc) and representative cages of cigar geometry ([Zr_6_(µ_3_-O)_2_(µ_2_-OH)_6_(Cp)_6_(bpdc)_3_]^2+^) and tetrahedron geometry ([Zr_12_(µ_3_-O)_4_(µ_2_-OH)_12_(Cp)_12_(bpdc)_6_]^4+^), where grey, yellow, and red represent carbon, zirconium, and oxygen atoms, respectively.

**Fig. 2:**
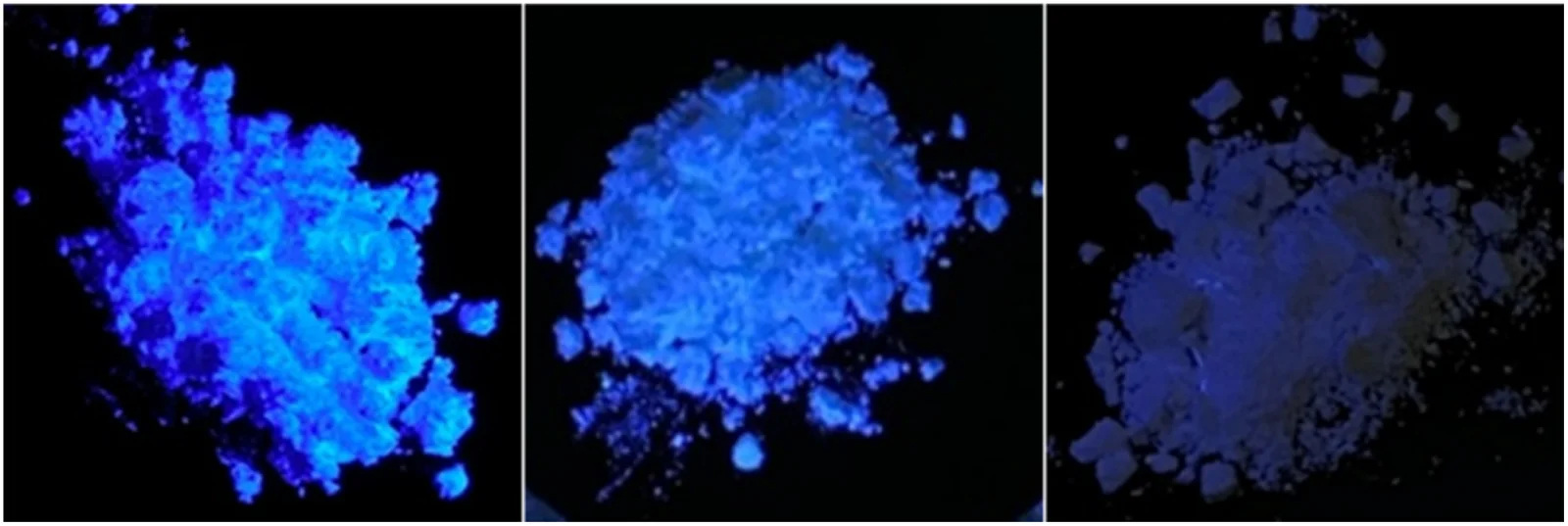
Solid samples of H_2_bpdc (left), [cage][Cl]-mix (center), and [cage][OTf]_2_-cig (right) individually under a 365 nm UV lamp.

These cages can be prepared using a variety of synthetic routes, most commonly involving the solvothermal reaction of ZrCp_2_Cl_2_ or zirconium acetate with the appropriate ligand in a suitable solvent.^39^ Cook and coworkers reported a benzoic acid cap-based strategy for accessing phase-pure tetrahedral cages,^40^ whereas Le Ouay and coworkers later described a separation procedure that enables isolation of either the tetrahedron or cigar forms for several cages as triflate salts.^41^ In our hands, however, perhaps due to subtle differences in reaction vessels, solvent water content, ambient temperature, or other experimental variables, we generally isolated either mixtures of the tetrahedron and cigar or exclusively the cigar species. Regardless of the synthetic route, we were unable to reproducibly isolate phase-pure tetrahedral material. This variability in speciation prompted us to examine how the relative distribution of cage geometries evolves over time and whether such changes influence fluorescence behavior.

Consistent with prior reports,^38,42^ we observed that the ratio of tetrahedron to cigar in solution decreases over time, reflecting the thermodynamically favored conversion between these species. Notably, however, the inherent fluorescence of a given cage solution did not decrease over time despite changes in speciation.

Accordingly, we chose to focus on the cigar geometry for this study, as phase-pure samples of this material could be obtained reliably and reproducibly. Here, the reaction of ZrCp_2_Cl_2_ with 4,4′-biphenyldicarboxylic acid in DMA, chloroform, and water at room temperature for 16 hours affords the mixed-phase cage ([cage][Cl]-mix), which was predominantly in the cigar phase, as an isolable solid in high yield. Starting from the chloride salt, ion metathesis proceeds cleanly: treatment of the cage with AgOTf affords the triflate salt of the cage ([cage][OTf]-mix) in quantitative yield. The triflate salt additionally exhibits enhanced solubility, particularly in alcoholic solvents. This further allows for separation of the cigar ([cage][OTf]_2_-cig) and tetrahedron phases *via* selective precipitation. Phase purity was confirmed by solution NMR spectroscopy and mass spectrometry, as evidenced by a single set of doublets in the former and the conspicuous absence of the 3 + *m*/*z* peak associated with the tetrahedral species in the latter (Fig. S3 and S8).

Notably, the chloride and triflate salts exhibit distinct fluorescence behavior in both solution and the solid state. To better compare the intrinsic photophysical behavior of the different cage salts, solution-state UV-vis and emission spectra were collected in methanol for H_2_bpdc and the chloride, triflate, nitrate, and bromide cage forms (Fig. S11–S13). The cage salts retain the characteristic biphenyl-based absorption but display counterion-dependent differences in emission intensity and maximum. In particular, the chloride cage is substantially more emissive than the triflate form in solution. These observations demonstrate that the counterion-dependent photophysical behavior is not restricted to the solid state, although the magnitude of the fluorescence differences is most pronounced following isolation of the corresponding cage salts.

To assess whether changes in cage speciation alone could account for the fluorescence differences, we monitored a chloride sample containing both tetrahedral and cigar species after storage at room temperature for one week. Over this period, the tetrahedron : cigar ratio decreased to the point that the tetrahedron was nearly absent, as confirmed by NMR and mass spectrometry (Fig. 3 and S1–S3). The sample remained strongly emissive throughout this process and even showed a modest increase in solution fluorescence (Fig. 3). These trends persist in the solid state. Qualitatively, H_2_bpdc, the chloride cage, and the triflate cage display solid-state emission following the trend ligand > chloride >> triflate. To rapidly and systematically assess these materials in the solid state, samples were loaded into a microplate and analyzed using a plate reader. Solid-state fluorescence intensities of 34 635, 3968, and 512 were measured for the ligand, [cage][Cl]-mix, and [cage][OTf]_2_-cig, respectively (*λ*_ex_ = 287 nm) (Table S2). Emission maxima for the ligand occurred at 410 nm, whereas the chloride and triflate salts exhibited a blue-shifted emission at 380 nm and 360 nm, respectively (Fig. S12).

**Fig. 3:**
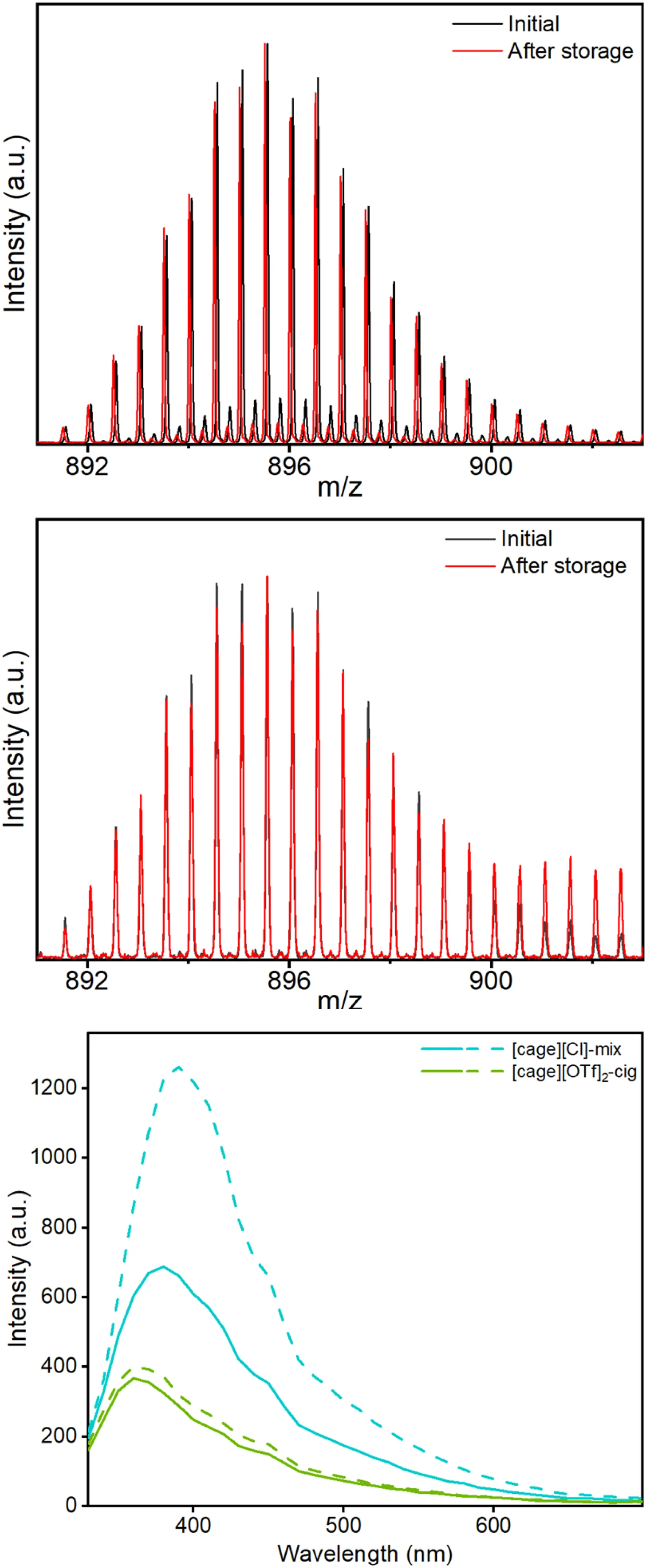
(Top) Mass spectra of [cage][Cl]-mix at 896 *m*/*z* with a ratio of tetrahedron : cigar decreasing after 1 week of storage. (Middle) Mass spectra of [cage][OTf]_2_-cig at 896 *m*/*z* with clear interpeak spacing of +2, after 1 week of storage. (Bottom) Emission spectra of [cage][Cl]-mix (teal) and [cage][OTf]_2_-cig (green) cages in methanol solutions initially made (solid trace) and after storing for 1 week (dashed trace).

To probe how added salts influence fluorescence, we combined solutions of the triflate cage with various salts in a microplate and then evaporated the solvent to afford solid materials. While this method does not guarantee complete ion metathesis as all ions remain in the wells and physical mixtures of salts cannot be unequivocally distinguished from metathesis products, it provides a rapid qualitative assay of anion-dependent fluorescence enhancement. Initial experiments focused on simple alkali metal halide salts, including NaBr, KBr, KF, KI, NaI, and LiCl (Fig. 4). A pronounced range of solid-state fluorescence intensities was observed, following the trend NaBr > KBr >> KI > LiCl > KF > NaI. Both NaBr and KBr produced substantial fluorescence enhancements. In contrast, the remaining salts had little to no effect on cage fluorescence. Notably, the introduction of chloride as various salts did not noticeably increase fluorescence under these screening conditions, which may reflect incomplete metathesis rather than a true absence of chloride-induced enhancement.

**Fig. 4:**
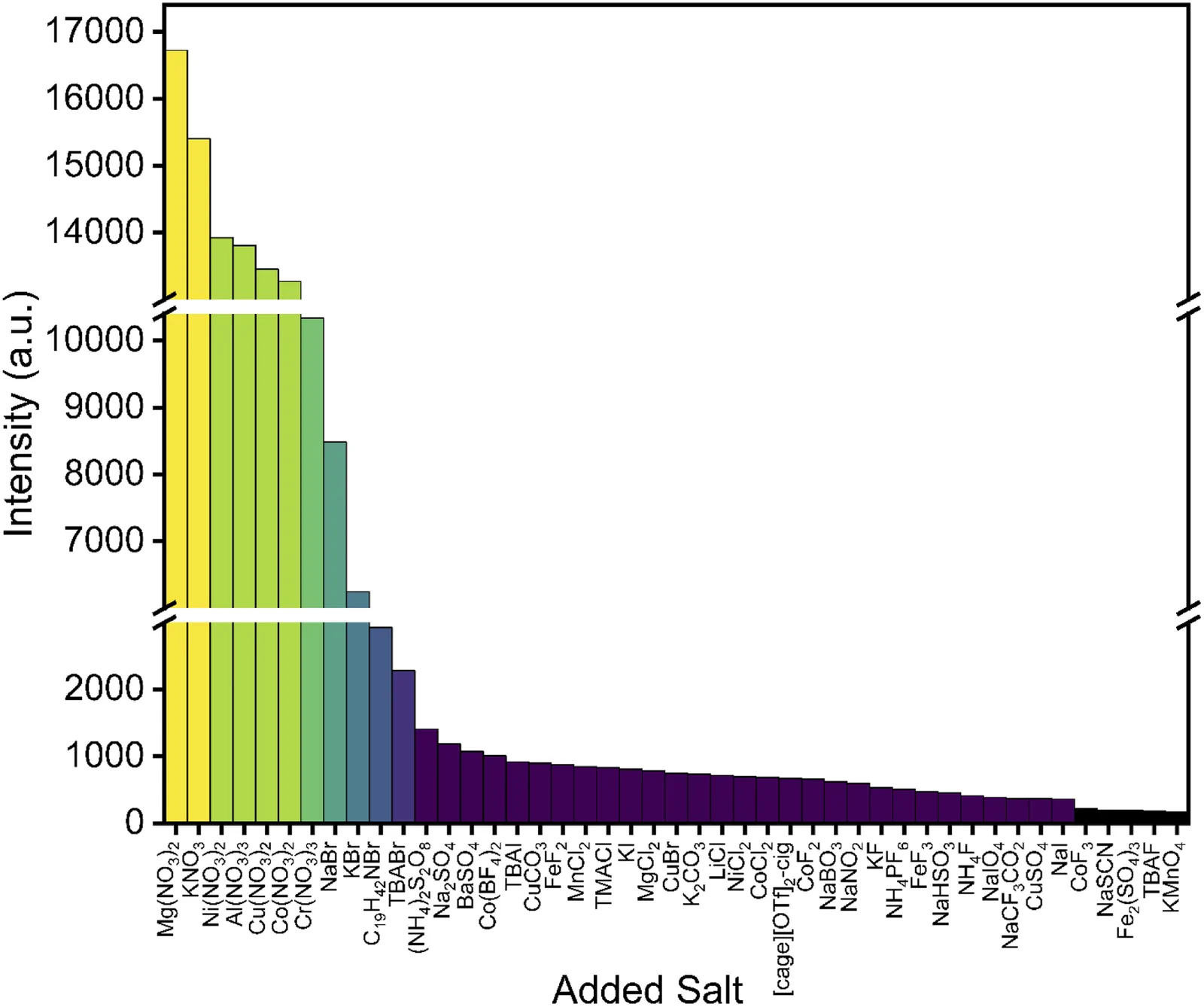
Resulting max fluorescence intensity values of [cage][OTf]_2_-cig introduced to various salts. The nitrate salts yielded the brightest fluorescence.

Encouraged by these qualitative results, we expanded the study to a broader set of 44 salt pairs. In these experiments, a solution of the triflate cage was pipetted into a microplate, followed by addition of an excess of AX, where A^*n*+^ included transition metals, alkali metals, alkaline earth metals, main group cations, and organic cations, and X^*n*−^ encompassed halides, oxyanions, and both organic and inorganic anions. Salts were prepared as methanolic solutions when possible. For salts that were not fully soluble, suspensions were transferred to the wells. Because this approach does not guarantee complete ion metathesis and may yield physical mixtures, the screen is a qualitative assessment of anion-dependent fluorescence enhancement. The broad salt screen demonstrates that most added salts result in fluorescence intensities at or below that of the parent triflate cage, indicating that fluorescence enhancement is not a general consequence of salt addition (Fig. 4 and Table S3). Taken together, this expanded survey reveals that the largest fluorescence enhancements are associated with nitrate and, to a lesser extent, bromide salts, with increases reaching values as high as 17 000 across multiple cations (Table S3). By contrast, transition metal halides, organic halides, and most other oxyanions generally result in minimal fluorescence enhancement relative to the parent triflate salt. Notably, BaSO_4_, a highly insoluble salt, yields fluorescence comparable to the parent cage, further supporting that simple solid mixing or ionic strength effects do not account for the observed enhancement behavior.

Closer inspection of the nitrate-containing entries within this same screen reveals a striking degree of consistency across cations. Emission spectra for Mg(NO_3_)_2_, Cu(NO_3_)_2_, Co(NO_3_)_2_, Al(NO_3_)_3_, Ni(NO_3_)_2_, Cr(NO_3_)_3_, and KNO_3_ all display nearly identical spectral profiles, with maxima centered near ∼400 nm (Fig. S15). Fluorescence intensities span approximately ∼10 000 to nearly 17 000, following the trend Mg > K > Ni ≈ Al > Cu > Co > Cr. Importantly, the persistence of strong emission across mono-, di-, and trivalent cations suggests that fluorescence enhancement is governed chiefly by nitrate identity rather than by cation electronics or oxidation state. The modest intensity variation within the nitrate series likely reflects subtle differences in ion pairing, lattice organization, or extent of metathesis under the qualitative screening conditions.

During treatment of the triflate cage with these nitrate salts, precipitation was observed upon mixing, consistent with strong cage–nitrate interactions. On the basis of these results, Mg(NO_3_)_2_·6H_2_O reacts with [cage][OTf]_2_-cig netting [cage][NO_3_]_2_ as an isolable solid. The resulting material exhibits identical ^1^H NMR resonances and mass spectrometry envelopes to those of the triflate cage, indicating preservation of the cage framework (Fig. S4 and S9). Importantly, the ^19^F NMR spectrum of the isolated solid shows no fluorine-containing species, confirming complete anion metathesis (Fig. S5).

A slow diffusion between 1 mL of 1000 µM [cage][OTf]_2_-cig layered onto 1 mL of 10 000 µM Mg(NO_3_)_2_·6H_2_O resulted in diffraction-quality needle-like crystals after 15 days. Analysis of the data confirms it adopts the expected structure comprising two clusters and three biphenyl ligands (Fig. 5). Similar to the published chloride and triflate salts for these types of cages,^21,29,38,41^ there are significant anion–cluster interactions in the solid state. For a given cage, a nitrate anion is hydrogen bonded to a cluster *via* the bridging OH groups. In contrast to triflate structures, the nitrate anions readily bridge cages (Fig. S19). This essentially results in a network structure where the anion bridges three separate cages. The bromide salt was also isolated and structurally characterized, providing an additional example in which counterion exchange preserves the cigar-shaped cage structure while producing a distinct anion-dependent hydrogen-bonding and packing environment (Fig. S26–S31).

**Fig. 5:**
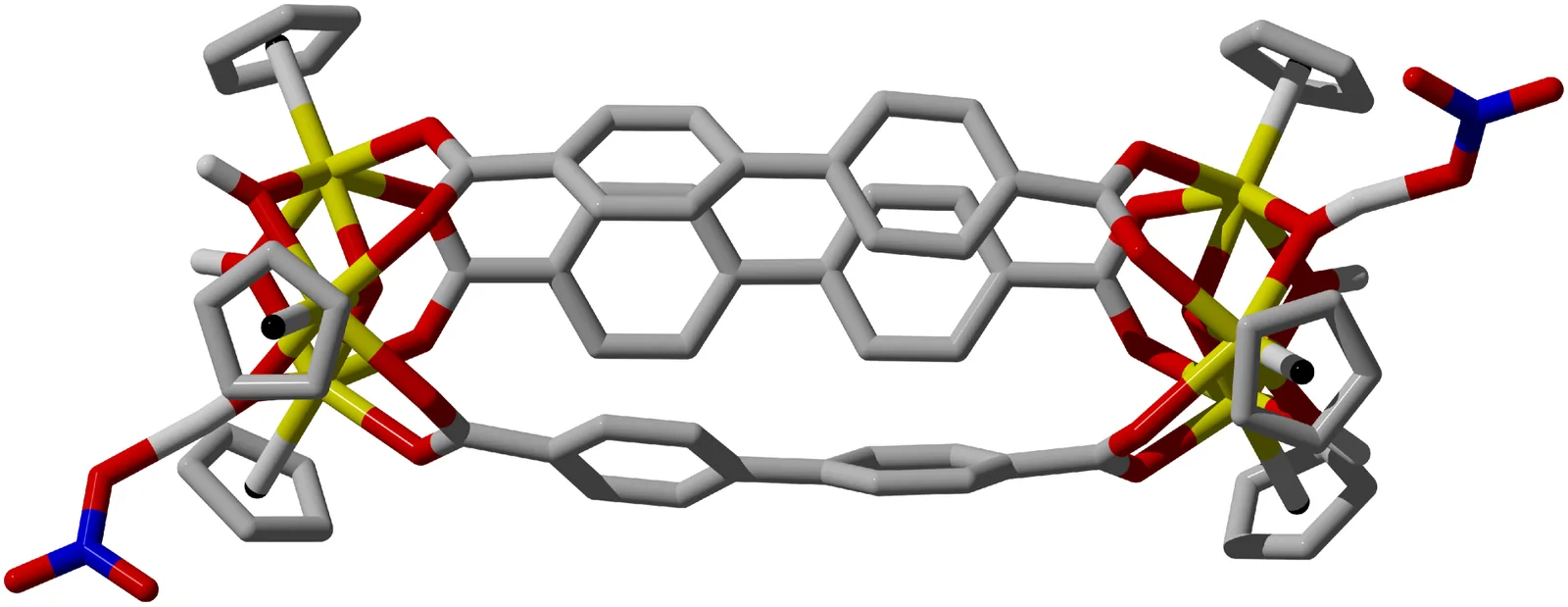
The crystal structure of [cage][NO_3_]_2_-cig featuring two trinuclear zirconium clusters, three bridging biphenyl linkers, and cluster-bound nitrate anions.

To establish that these materials retain accessible porosity following counterion exchange, CO_2_ adsorption measurements were collected at 195 K for the chloride, triflate, nitrate, and bromide cage salts (Fig. S10 and Table S1). All four materials exhibit measurable gas uptake, with BET surface areas of 216, 131, 133, and 66 m^2^ g^−1^ for [cage][Cl]-mix, [cage][OTf]_2_-cig, [cage][NO_3_]_2_-cig, and [cage][Br]_2_-cig, respectively. Thus, exchange of the external counterion alters the accessible surface area but does not eliminate porosity. The similar BET surface areas of the triflate and nitrate forms are particularly notable because these materials display substantially different fluorescence behavior, indicating that the enhanced emission of the nitrate salt cannot be attributed simply to a large change in overall accessible porosity.

To further probe the relationship between nitrate loading and fluorescence enhancement, we performed a concentration-dependent study in which the amount of Mg(NO_3_)_2_·6H_2_O was varied (250, 750, 1000, 5000 up to 100 000 µM in increments of 5000) while the amount of [cage][OTf]_2_-cig remained constant (Table S4). Cage-only wells were included for calibration. After solvent removal, fluorescence intensities were measured and normalized relative to the absorbance per well.

The resulting data reveal a concentration- and stoichiometry-dependent fluorescence response for Mg(NO_3_)_2_ (Fig. S16, S17 and Table S5). High emission intensity occurs with even small amounts of Mg(NO_3_)_2_ relative to the cage. As the salt loading decreases, fluorescence generally diminishes, approaching the weak emission of the parent triflate cage at very low nitrate levels (≤0.03 equiv). These results indicate that fluorescence enhancement depends strongly on nitrate loading, with a threshold nitrate : cage ratio required to generate the emissive state. Notably, significant fluorescence enhancement is already observed at approximately 0.12 equivalents of Mg(NO_3_)_2_ (0.24 equivalents of nitrate), corresponding to only 10% triflate-to-nitrate metathesis per cage. Thus, even limited nitrate incorporation is sufficient to produce a large fluorescence increase.

## Conclusions

We have shown that fluorescence in biphenyl-functionalized zirconium porous cages can be tuned through counterion exchange. While the chloride salt exhibits strong emission and the triflate salt is largely non-emissive, broad salt screening shows that fluorescence recovery is highly anion-dependent, with nitrate salts producing the most pronounced enhancement across a range of cations. Isolation and characterization of [cage][NO_3_]_2_-cig confirm that nitrate exchange preserves the cage structure while removing triflate completely, and single-crystal diffraction suggests that nitrate-specific cage–anion interactions help promote the strongly emissive state. Together, these results establish counterion identity as a useful handle for tuning the photophysical behavior of zirconium porous cages and provide a framework for studying counterion-controlled fluorescence in porous cage salts.

## Author contributions

This project was conceptualized by R. M. Sabatino, M. N. Morey, and E. D. Bloch. Syntheses, anion exchanges, and characterization were performed by R. M. Sabatino. Photophysical studies were conducted by R. M. Sabatino. Crystal structures were collected and resolved by A. Lovstedt for CCDC 2539471 and assisted R. M. Sabatino for CCDC 2583584. The entire project was supervised by E. D. Bloch, and manuscript was written by R. M. Sabatino and E. D. Bloch.

## Conflicts of interest

There are no conflicts to declare.

## Supplementary Material

SC-OLF-D6SC06722K-s001

SC-OLF-D6SC06722K-s002

## Data Availability

CCDC 2583584 and 2539471 contain the supplementary crystallographic data for this paper.^43*a*,*b*^ The data supporting this article have been included as part of the supplementary information (SI). Supplementary information is available. See DOI: https://doi.org/10.1039/d6sc06722k.
