# Supplementary material for: Anion-specific fluorescence enhancement via counterion modulation in biphenyl-based porous cage salts
Source: Chem Sci. 2026 Oct 22. Online ahead of print. doi: 10.1039/d6sc06722k (PMC13688255; doi:10.1039/d6sc06722k)
Supplement: Supplementary file 1 [file SC-OLF-D6SC06722K-s001.pdf]

Supplementary Information (SI) for Chemical Science.
This journal is © The Royal Society of Chemistry 2026

Electronic Supplementary Information

Anion-specific fluorescence enhancement via counterion modulation in biphenyl-based porous cage salts

Rachel M. Sabatino,[a] Merissa N. Morey,[a] Alex Lovstedt[a] and Eric D. Bloch*[a]

[a]Department of Chemistry, Indiana University, Bloomington, IN 47405, United States

Email: edbloch@iu.edu

Table of Contents

A. General Considerations S3
B. Cage Synthesis S4
C. Procedures for Anion Exchange Studies S5
D. Characterization S7
E. Fluorescence-based Anion Recognition Data S20
F. Single Crystal X-ray Diffraction S27
G. References S43

A. General Considerations

Materials Methanol (MeOH), chloroform, toluene, diethyl ether, *N,N*-dimethylacetamide (DMA), zirconocene dichloride, 4,4'-biphenyldicarboxylic acid (H_2bpdc), silver trifluoromethanesulfonate (AgOTf), magnesium nitrate hexahydrate ($Mg(NO_3)_2$), copper(II) nitrate hemipentahydrate ($Cu(NO_3)_2$), cobalt(II) nitrate hexahydrate ($Co(NO_3)_2$), aluminum(III) nitrate nonahydrate ($Al(NO_3)_3$), nickel(II) nitrate hexahydrate ($Ni(NO_3)_2$), chromium(III) nitrate nonahydrate ($Cr(NO_3)_3$), sodium bromide (NaBr), potassium bromide (KBr), potassium nitrate (KNO_3), hexadecyltrimethylammonium bromide ($C_{19}H_{42}NBr$), tetrabutylammonium bromide (TBABr), ammonium persulfate ($(NH_4)_2S_2O_8$), barium sulfate ($BaSO_4$), sodium sulfate (Na_2SO_4), potassium carbonate (K_2CO_3), copper(II) carbonate ($CuCO_3$), cobalt(II) fluoride (CoF_2), cobalt(II) tetrafluoroborate hexahydrate ($Co(BF_4)_2$), sodium nitrite ($NaNO_2$), potassium fluoride (KF), ammonium hexafluorophosphate (NH_4PF_6), copper(II) sulfate pentahydrate ($CuSO_4$), tetramethylammonium chloride (TMACl), sodium bisulfite ($NaHSO_3$), lithium chloride (LiCl), ammonium fluoride (NH_4F), magnesium chloride hexahydrate ($MgCl_2$), potassium iodide (KI), cobalt(II) chloride hexahydrate ($CoCl_2$), sodium periodate ($NaIO_4$), sodium perborate tetrahydrate ($NaBO_3$), manganese(II) chloride tetrahydrate ($MnCl_2$), sodium thiocyanate (NaSCN), iron(III) sulfate ($Fe_2(SO_4)_3$), sodium iodide (NaI), cobalt(III) fluoride (CoF_3), tetrabutylammonium fluoride trihydrate (TBAF), sodium trifluoroacetate ($NaCF_3CO_2$), copper(I) bromide (CuBr), potassium permanganate ($KMnO_4$), and iron(III) fluoride (FeF_3) were obtained from commercial vendors and used without further purification.

NMR ^1H and ^{19}F NMR spectra were obtained using a Bruker 500 MHz spectrometer and data obtained was processed in MestReNova NMR processor software.

Liquid chromatography-mass spectrometry (LCMS) LCMS experiments were run on a Waters Synapt G2S HDMS. Data was analyzed using MassLynx software.

Surface Area Analysis Gas adsorption measurements were measured on a Micromeritics Tristar II PLUS using CO_2 at 195 K. Before measuring, samples were activated overnight at room temperature under dynamic vacuum.

UV-vis Spectroscopy UV-vis spectra were conducted using an AvaSpec-ULS2048CL-EVO spectrometer.

Fluorescence Solution fluorescence data were recorded using a photoluminescence spectrometer (FLS-1000) from Edinburgh Instruments upon excitation at 290 nm with a bandwidth of 4 nm for excitation and emission. Rapid fluorescence assay measurements were conducted in black clear-bottom 96-well microplates using a BioTek Synergy Neo2 Plate Reader with excitation at 287 nm and emission starting at 330 nm at room temperature.

B. Cage Synthesis

Synthesis of [cage][Cl]-mix

Synthesis was adapted from literature.[1] In a 500 mL RBF, zirconocene dichloride (4.0 g, 13.7 mmol) and H_2bpdc (2.0 g, 8.3 mmol) were suspended in 100 mL DMA and sonicated for 10 minutes. Next, 50 mL chloroform and 30 mL deionized water were added and the flask was capped before vigorously stirring for 16 h at room temperature, resulting in the formation of a white solid. The solid was collected by vacuum filtration using a fine fritted glass funnel, washed with two-times 20 mL DMA then three-times 50 mL diethyl ether, and dried under reduced pressure for several hours. The synthesis yielded 4.13 g of product. Mass spectrometry data is shown below in Figure S6.

Synthesis of [cage][OTf]-mix

Synthesis was adapted from literature.[1] AgOTf (0.75 g, 2.92 mmol) was introduced to a 500 mL RBF wrapped with aluminum foil and dissolved in 150 mL MeOH and 25 mL toluene. [cage][Cl]-mix (2.5 g) was then added to the flask while stirring and suspension was kept vigorously stirring in the dark for three days. After this, the suspension was separated via vacuum filtration using a fine fritted glass funnel to isolate silver byproduct. Then, the filtrate was filtered through a PTFE syringe filter into a new RBF yielding a clear, colorless filtrate. An additional 50 mL MeOH were used to wash the solid and combine with the filtrate. After this, 150 mL toluene was added to the filtrate, and the solution was evaporated in a rotary evaporator until the volume of solvent was about ~50 mL remaining. Then 100 mL toluene was added to the flask, and the volume was reduced again to ~25 mL remaining. At this point, [cage][OTf]-mix had formed as a white precipitate and was collected by vacuum filtration. The solid product was washed with 25 mL toluene and two-times 50 mL diethyl ether and dried under reduced pressure for several hours. The synthesis yielded 2.28 g of product. ^{1}H NMR and mass spectrometry characterization data are shown below in Figure S1 and S7, respectively.

Synthesis of $[Zr_6(\mu_3\text{-}O)_2(\mu_2\text{-}OH)_6(Cp)_6(bpdc)_3]OTf_2$ ([cage][OTf]$_2$-cig)

Synthesis was adapted from literature to isolate pure cigar phase cage.[1] In a 500 mL RBF, [cage][OTf]-mix (1.5 g) was suspended in 50 mL MeOH. While stirring, 300 mL toluene was slowly added in 50 mL increments, resulting in the formation of a white solid. The mixture was separated via vacuum filtration and washed two-times 50 mL diethyl ether. After this, the filtrate was periodically poured onto a large watch glass and evaporated to dryness, resulting in the formation of [cage][OTf]$_2$-cig as a white solid. The solid was resuspended in minimal amount of MeOH then reprecipitated by the addition of 75 mL diethyl ether, before being collected via vacuum filtration washed two-times 50 mL diethyl ether and dried under reduced pressure for several hours. The synthesis yielded 354 mg

of product. ^1H NMR and mass spectrometry characterization data are shown below in Figure S2, S3,and S8, respectively.

C. Procedures for Anion Exchange Studies

NMR Sample Preparation of [cage][NO_3]$_2$-cig

In a 4 mL vial, 400 μL of 1,000 μM [cage][OTf]$_2$-cig MeOH solution was combined with 400 μL of 10,000 μM $Mg(NO_3)_2$ MeOH solution, yielding a white precipitate. The MeOH mother liquor was decanted off, and the precipitate was dried before redissolving in ~1 mL of MeOD. Spectra for ^1H and ^{19}F NMR shown below in Figure S4 and S5, respectively.

LCMS Sample Preparation of [cage][NO_3]$_2$-cig

In a 4 mL vial, 400 μL of 1,000 μM [cage][OTf]$_2$-cig MeOH solution was combined with 400 μL of 10,000 μM $Mg(NO_3)_2$ MeOH solution, yielding a white precipitate. The MeOH mother liquor was decanted off, and the precipitate was redissolved in 10 mL of fresh MeOH. Data is shown below in Figure S9.

Crystallization of [cage][NO_3]$_2$-cig and [cage][Br]$_2$-cig

In an NMR tube, 1 mL of 1,000 μM [cage][OTf]$_2$-cig MeOH solution was layered on top of 1 mL of 10,000 μM $Mg(NO_3)_2$ MeOH solution for slow diffusion. After 15 days, diffraction-quality needle-like crystals were obtained for SCXRD. In a 2 mL vial, 1 mL of 1,000 μM [cage][OTf]$_2$-cig MeOH solution was layered on top of 1 mL of 10,000 μM $C_{19}H_{42}NBr$ MeOH solution for slow diffusion. After roughly a month, a diffraction-quality block crystal was obtained for SCXRD. Crystal structures and data collection information are provided under Section F on page S27.

UV-vis Absorption Measurements

5 μM MeOH solutions of H_2bpdc (3 equiv.), [cage][Cl]-mix, [cage][OTf]$_2$-cig, [cage][NO_3]$_2$-cig, and [cage][Br]$_2$-cig were prepared for absorption on AvaSpec-ULS2048CL-EVO spectrometer and data is shown below in Figure S11.

Solution Fluorescence

5 μM MeOH solutions of H_2bpdc (3 equivalents), [cage][Cl]-mix, and [cage][OTf]$_2$-cig were prepared for emission measurements on FLS-1000 and data is shown below in Figure S12 and S13.

Rapid Fluorescence Assays

<u>Fluorescence Emission Pre and Post Storage</u>

2600 μM MeOH solutions of H_2bpdc, [cage][Cl]-mix, and [cage][OTf]$_2$-cig were prepared in 20 mL vials. The solutions were added as triplicates to a 96-well plate directly before

collecting data to ensure no solvent had evaporated beforehand. The vials were stored in a drawer for 1 week then remeasured the solution fluorescence data of the samples. Data is provided below in Table S3 and Figure S14.

Broad Salt Screening

Solutions of [cage][OTf]$_2$-cig (112.3 mg, 1 eq., 20 mL MeOH, 0.00269 M) and 44 different salts (10 eq., 10 mL MeOH, ~0.027 M) were prepared in vials. The 96-well plate contained 150 µL [cage][OTf]$_2$-cig with 50 µL salt for each well in addition to 6 wells that only had [cage][OTf]$_2$-cig for calibration. Solvent was evaporated off before collecting fluorescence data of the solids at room temperature. Data is provided below in Table S4 and Figure S15.

Concentration Dependence of $Mg(NO_3)_2 \cdot 6H_2O$

Serial dilutions of 0.100776 M $Mg(NO_3)_2$ stock solution with MeOH were conducted to prepare several concentrations: 250, 750, 1,000, 5,000, 10,000, 15,000, 20,000, 25,000, 30,000, 35,000, 40,000, 45,000, 50,000, 55,000, 60,000, 65,000, 70,000, 75,000, 80,000, 85,000, 90,000, 95,000, 100,000 µM. A 20 mL MeOH solution of [cage][OTf]$_2$-cig (113.1 mg, 0.05451 mmol, 2,700 µM) was prepared before diluting to 1,000 µM. The well plate contained 150 µL [cage][OTf]$_2$-cig with 50 µL $Mg(NO_3)_2 \cdot 6H_2O$ for each cell in ascending order in addition to 3 wells that only had [cage][OTf]$_2$-cig for calibration. Solvent was evaporated off before collecting fluorescence data of the solids at room temperature. Data is provided below in Table S5 and Figures S16 and S17.

D. Characterization

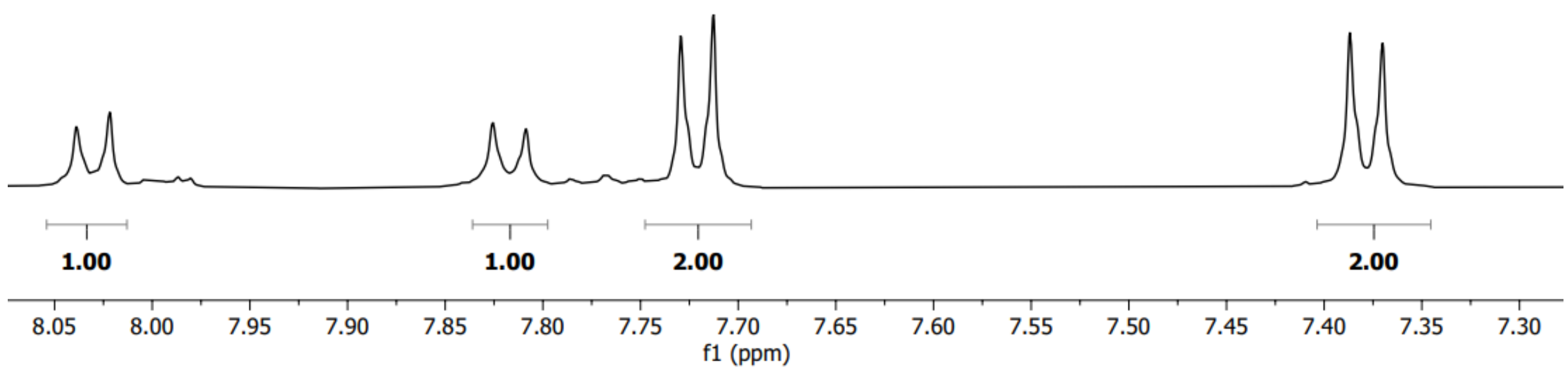

Figure S1. ^{1}H NMR in MeOD for [cage][OTf]-mix to determine tetrahedron:cigar ratio. The two doublets at 7.38 and 7.72 ppm represent the bpdc ligand configured in cigar geometry. The two downfield doublets at 7.82 and 8.03 ppm represent the bpdc ligand configured in tetrahedron geometry. There is a 1:2 ratio between the two geometries.

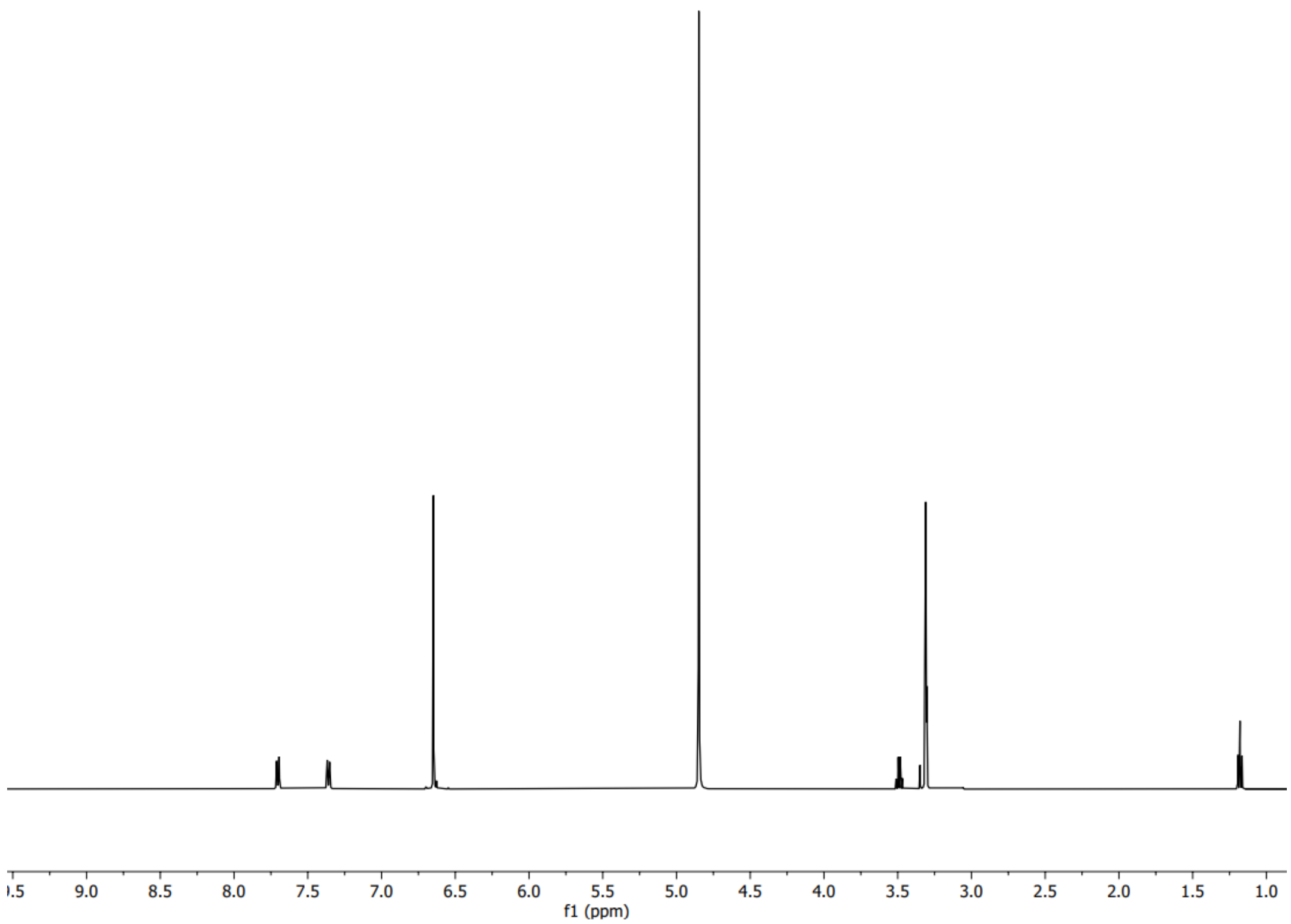

Figure S2. ^{1}H NMR in MeOD for [cage][OTf]$_2$-cig. The peak around 6.65 ppm corresponds to the cyclopentadienyl rings on the zirconium nodes of the cage. The two doublets at 7.36 and 7.70 ppm represent the bpdc linkers. Solvent peaks are observed at 4.85 s for water, and 3.49 q and 1.18 t for diethyl ether.

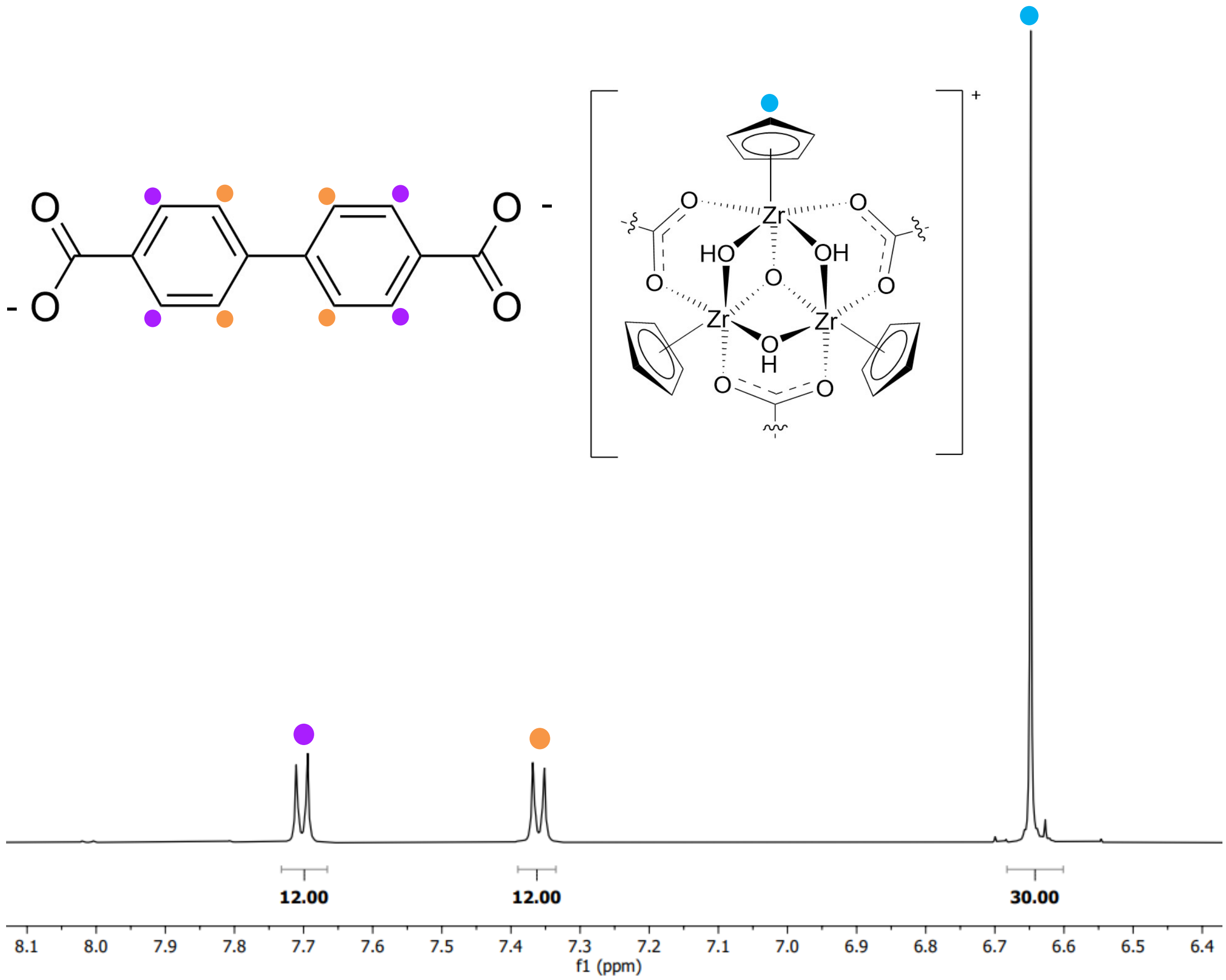

Figure S3. ^{1}H NMR in MeOD for [cage][OTf]$_2$-cig zoomed in to key peaks. The peak around 6.65 ppm corresponds to the cyclopentadienyl rings on the zirconium nodes of the cage that integrates to 30.00 since there are two nodes. The two doublets at 7.36 and 7.70 ppm represent the bpdc ligands with an integration of 12.00 for the three linkers in the cigar cage. No set of doublets corresponding to tetrahedral geometry is observed.

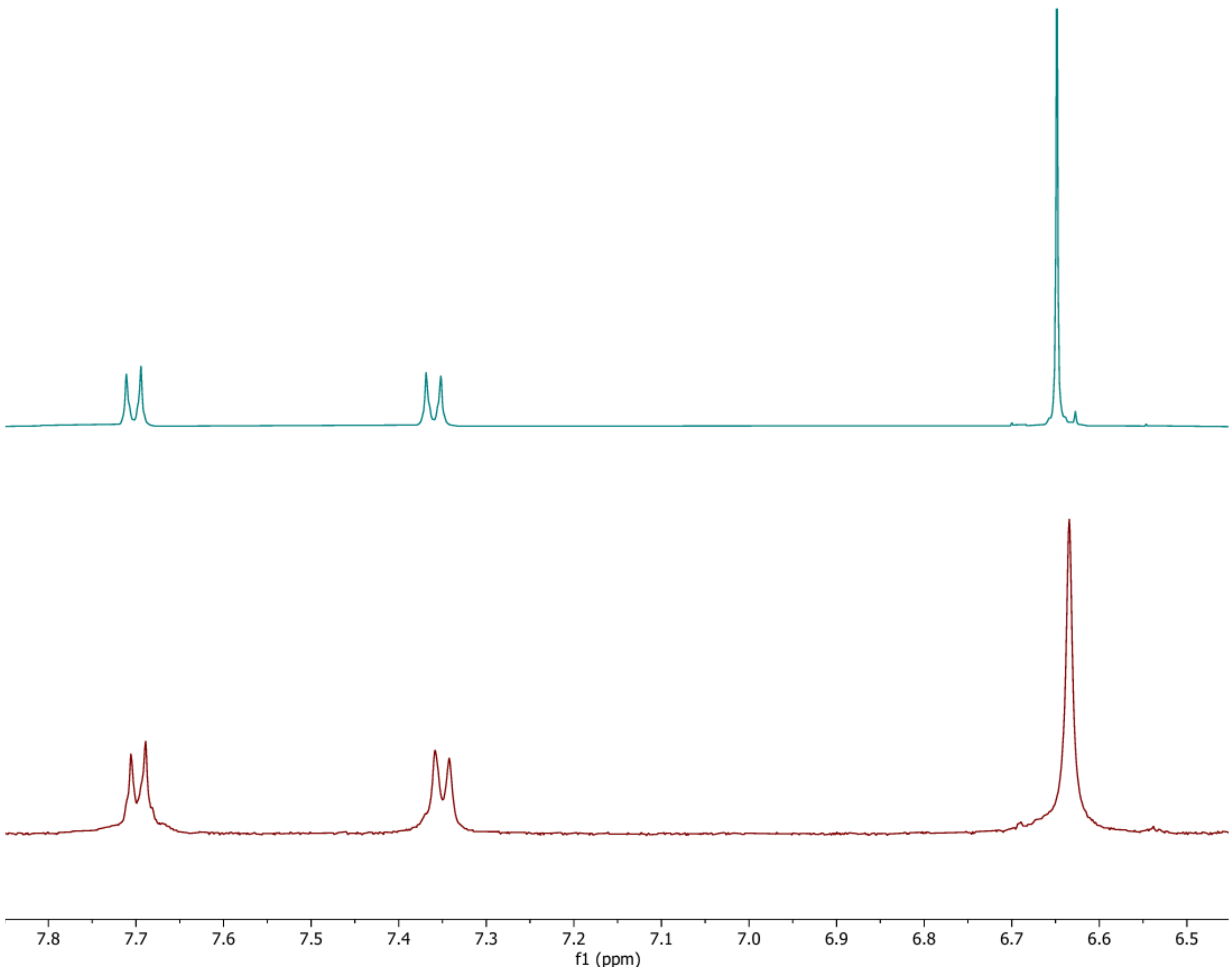

Figure S4. ^{1}H NMR in MeOD for [cage][OTf]$_2$-cig (blue trace) and [cage][NO_3]$_2$-cig (red trace), which was obtained from the precipitation of 1000 μM [cage][OTf]$_2$-cig with 10,000 μM $Mg(NO_3)_2$ and redissolved in MeOD. The peak around 6.64 ppm corresponds to the cyclopentadienyl rings on the zirconium nodes of the cage. The two doublets at 7.35 and 7.70 ppm represent the bpdc linker of the cigar cage.

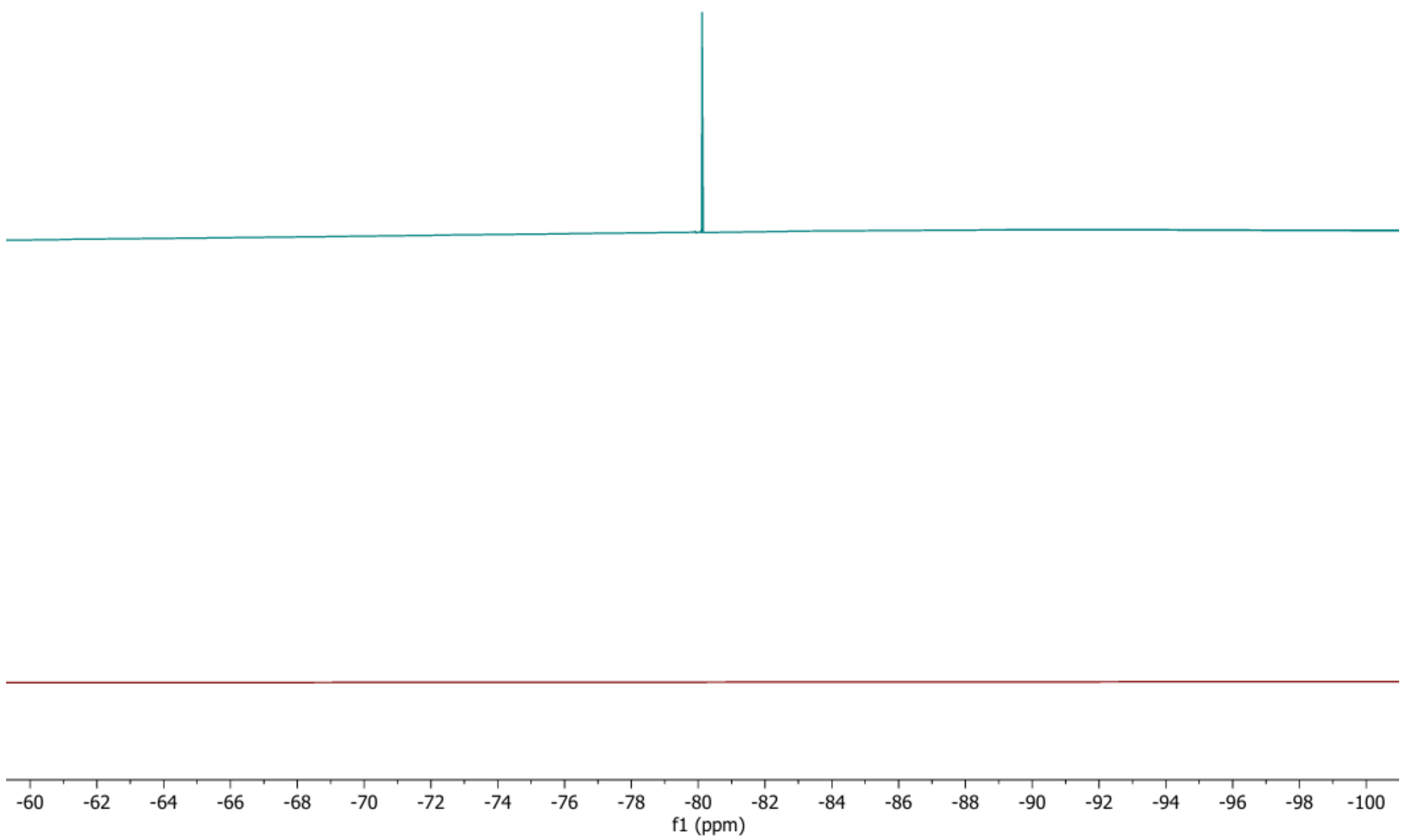

Figure S5. ^{19}F NMR in MeOD for [cage][OTf]$_2$-cig (blue trace) and [cage][NO$_3$]$_2$-cig (red trace), which was obtained from the precipitation of 1000 µM [cage][OTf]$_2$-cig with 10,000 µM $Mg(NO_3)_2$ and redissolved in MeOD. The peak around 6.64 ppm corresponds to the cyclopentadienyl rings on the zirconium nodes of the cage. The two doublets at 7.35 and 7.70 ppm represent the bpdc linker of the cage.

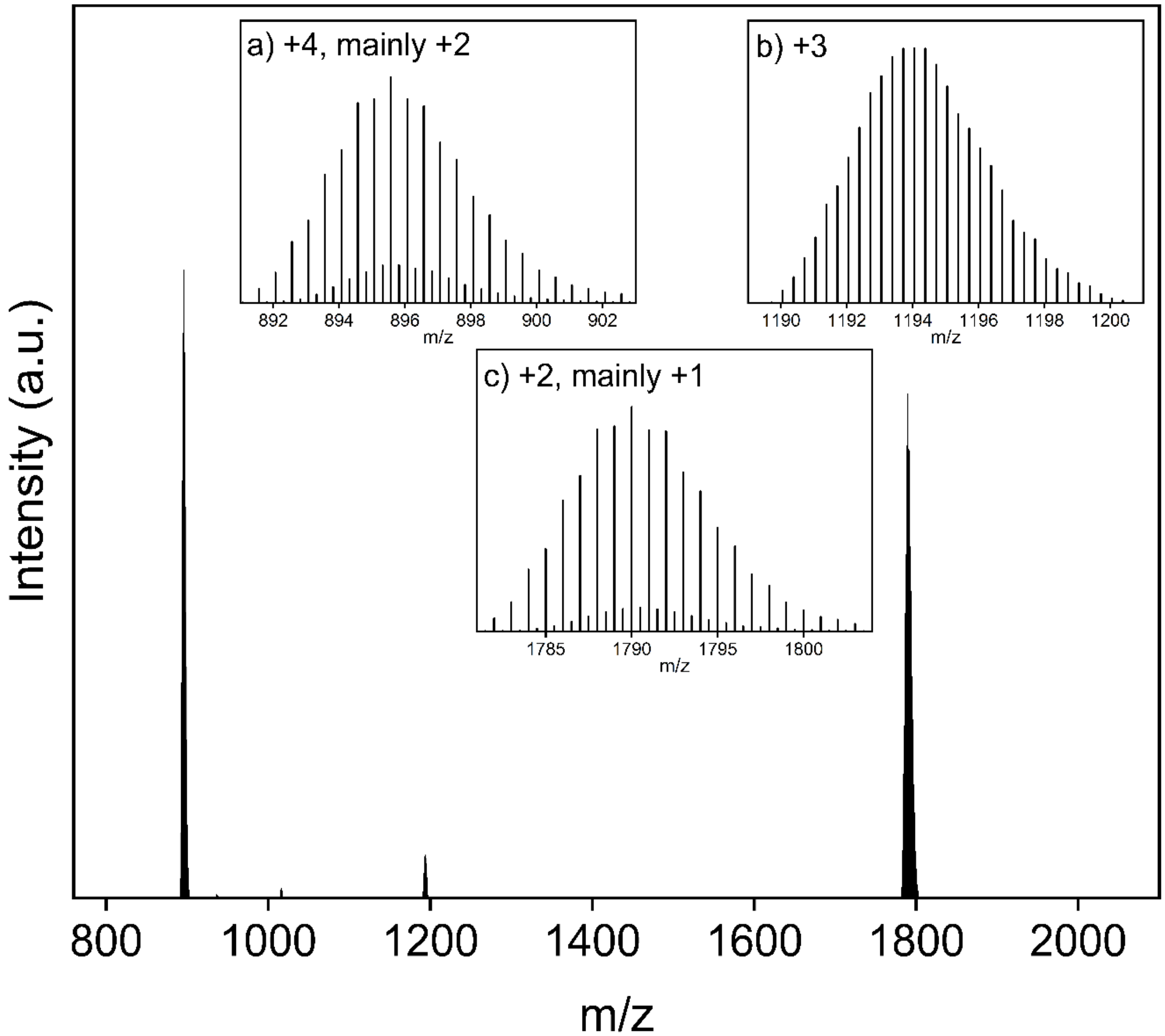

Figure S6. LCMS spectra for [cage][Cl]-mix. Due to the presence of 0.25 and 0.5 interpeak spacing at 896 and 1790 m/z, the chloride salt of the cage is a mixture of tetrahedron and cigar phases. The +2 peak at 896 m/z and +1 peak at 1790 m/z are representative of cigar phase. The +4, +3, and +2 peaks at 896, 1194, and 1790 m/z respectively, correspond to tetrahedron phase. Since the cigar phase peaks are more predominant than the tetrahedron phase, the cage is primarily cigar.

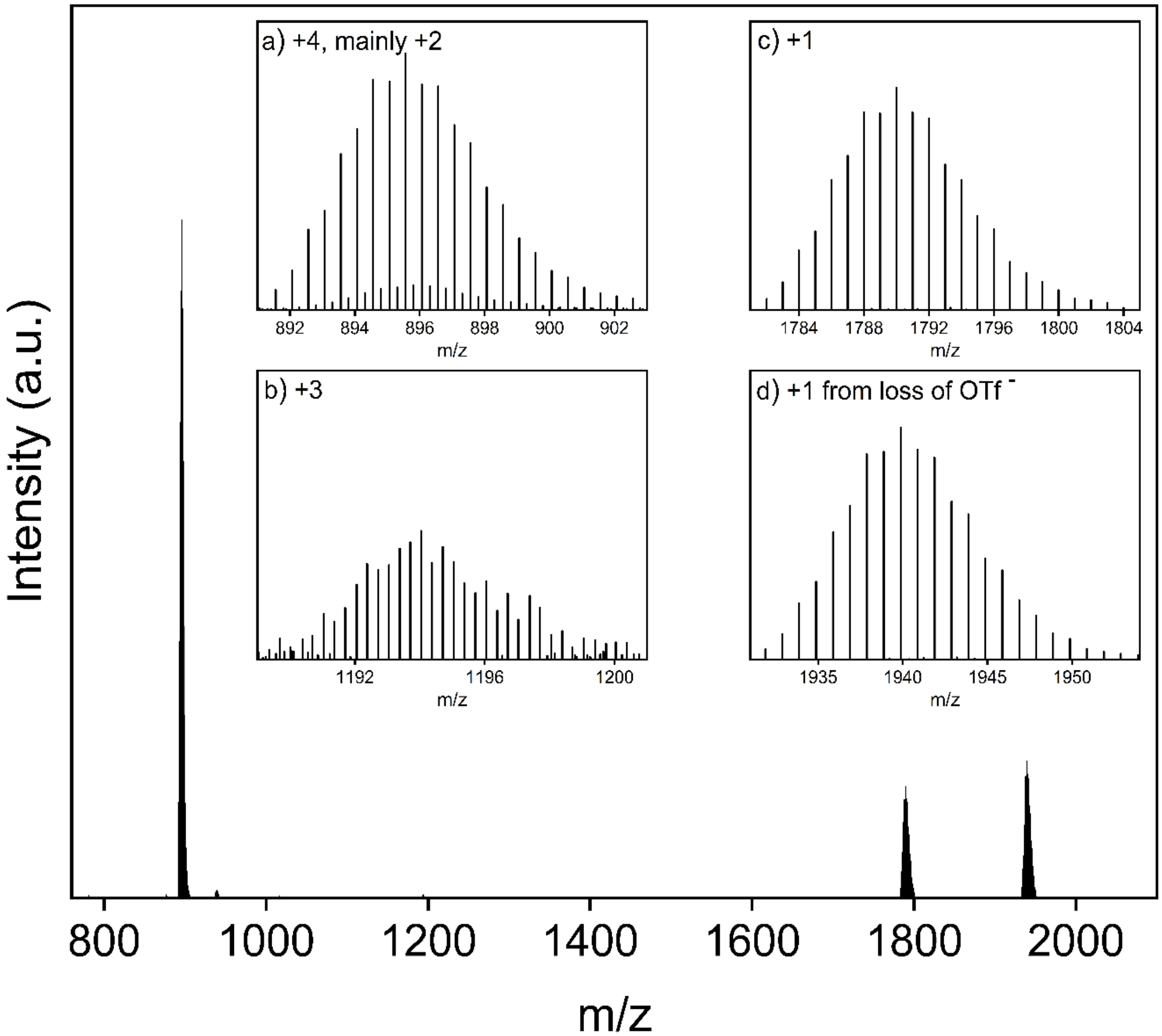

Figure S7. LCMS spectra for [cage][OTf]-mix. Due to the presence of 0.25 and 0.5 interpeak spacing at 896 m/z, the triflate salt of the cage is a mixture of tetrahedron and cigar phases. The +2 peak at 896 m/z and +1 peaks for 1790 and 1940 m/z are representative of cigar phase. The +4 and +3 peaks at 896 and 1194 m/z respectively, correspond to tetrahedron phase. Since the cigar phase peaks are more predominant than the tetrahedron phase, the cage is primarily cigar.

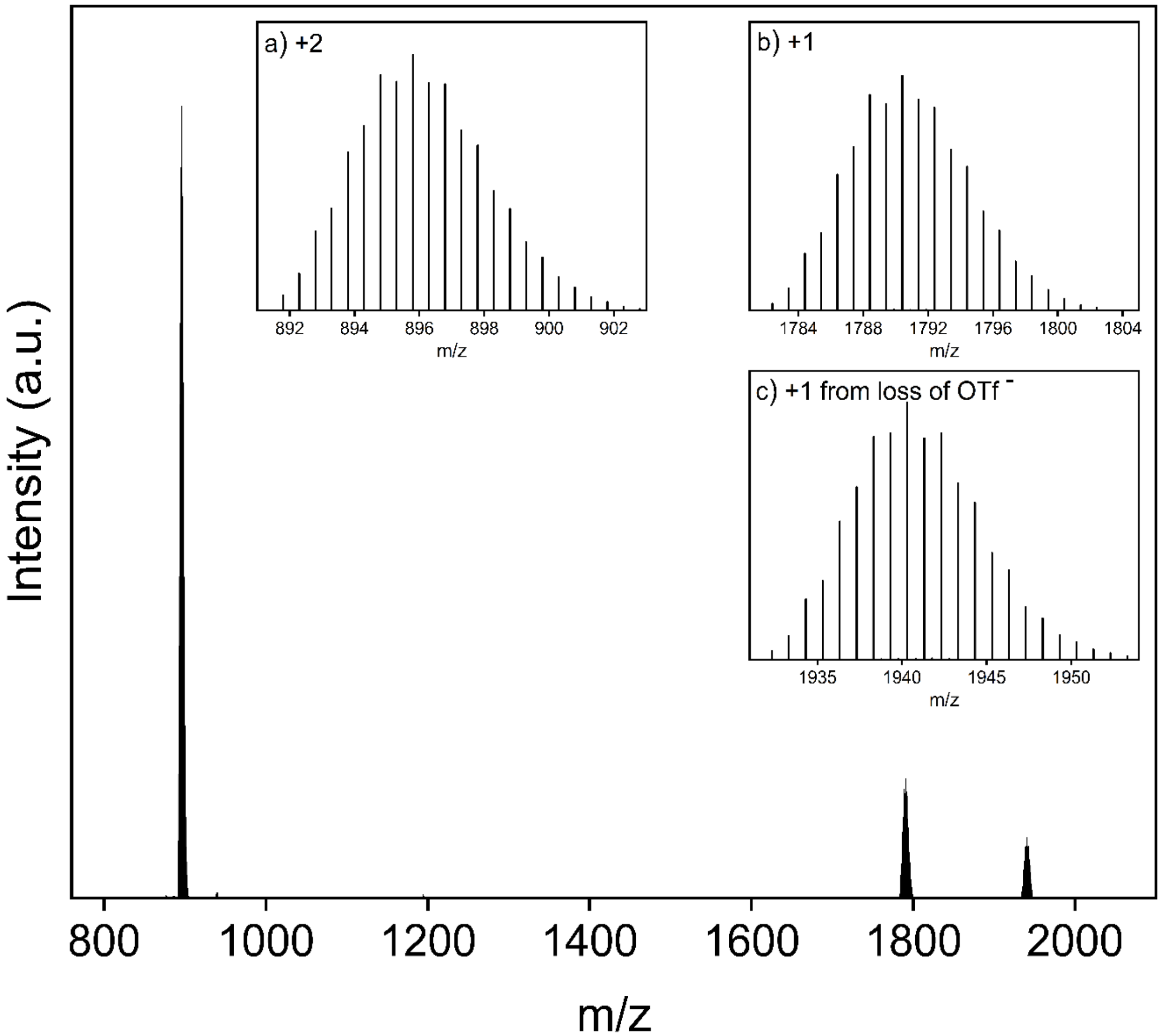

Figure S8. LCMS spectra for [cage][OTf]$_2$-cig. The presence of +2 and +1 peaks, around 896 and 1791 m/z respectively, indicates the cage's phase is cigar. The +1 peak near 1940 m/z corresponds to the loss of one triflate counterion.

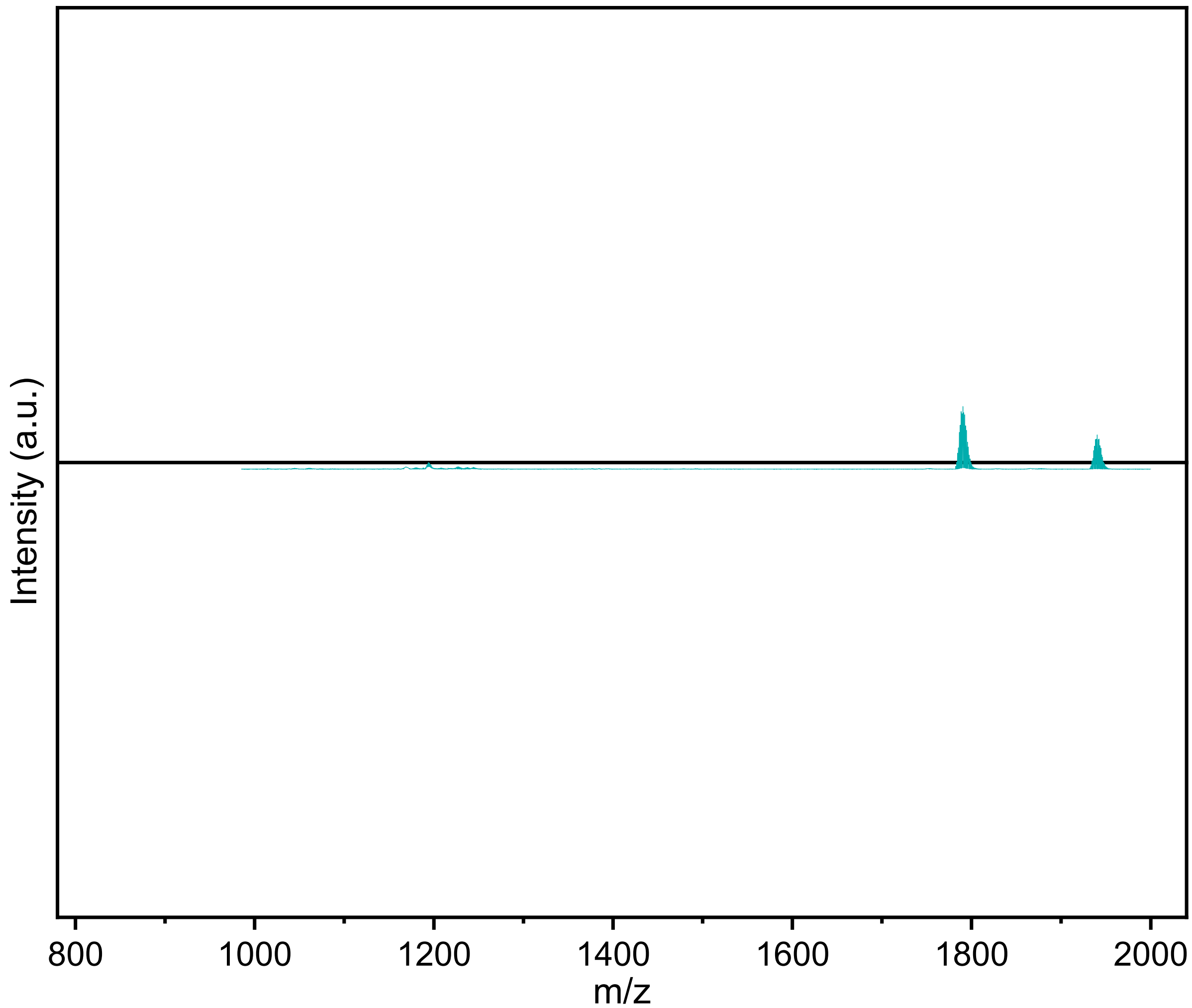

Figure S9. LCMS spectra of [cage][OTf]$_2$-cig (blue) and redissolved precipitate [cage][NO$_3$]$_2$-cig (red) that was obtained from combining methanolic solutions of 1000 µM [cage][OTf]$_2$-cig with 10,000 µM $Mg(NO_3)_2$.

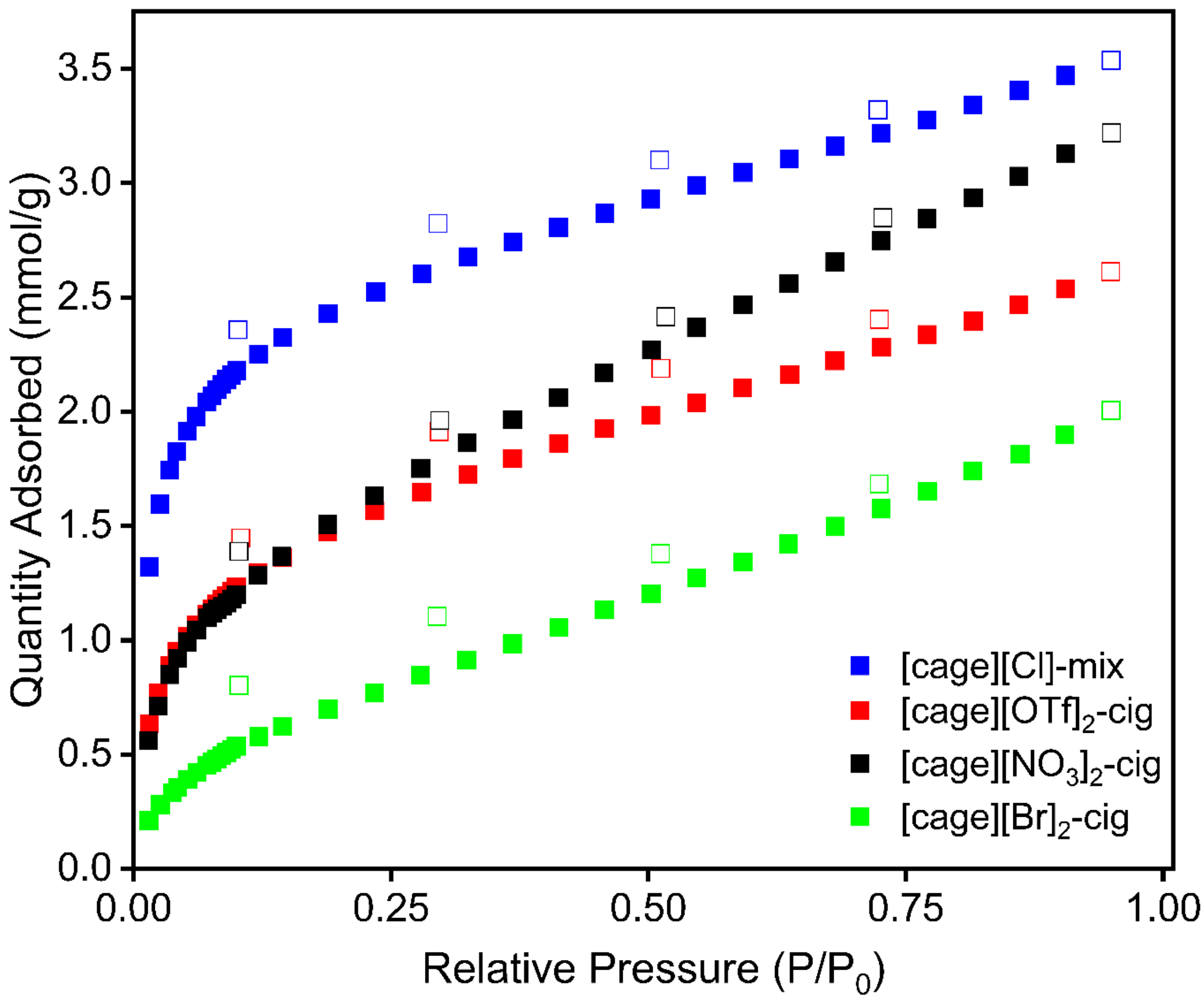

Figure S10. CO_2 adsorption isotherms of [cage][Cl]-mix (blue), [cage][OTf]$_2$-cig (red), [cage][NO$_3$]$_2$-cig (black), and [cage][Br]$_2$-cig (green) measured at 195 K. Solid points are representative of adsorption, while outline points are representative of desorption.

Table S1. Surface areas from CO_2 gas adsorption at 195 K.

Sample	BET (m²/g)	Langmuir (m²/g)
[cage][Cl]-mix	216.2 ± 0.95	441.6 ± 8.09
[cage][OTf]$_2$-cig	131.1 ± 0.53	370.4 ± 9.80
[cage][NO$_3$]$_2$-cig	132.8 ± 1.06	566.1 ± 16.2
[cage][Br]$_2$-cig	65.5 ± 1.24	607.9 ± 47.5

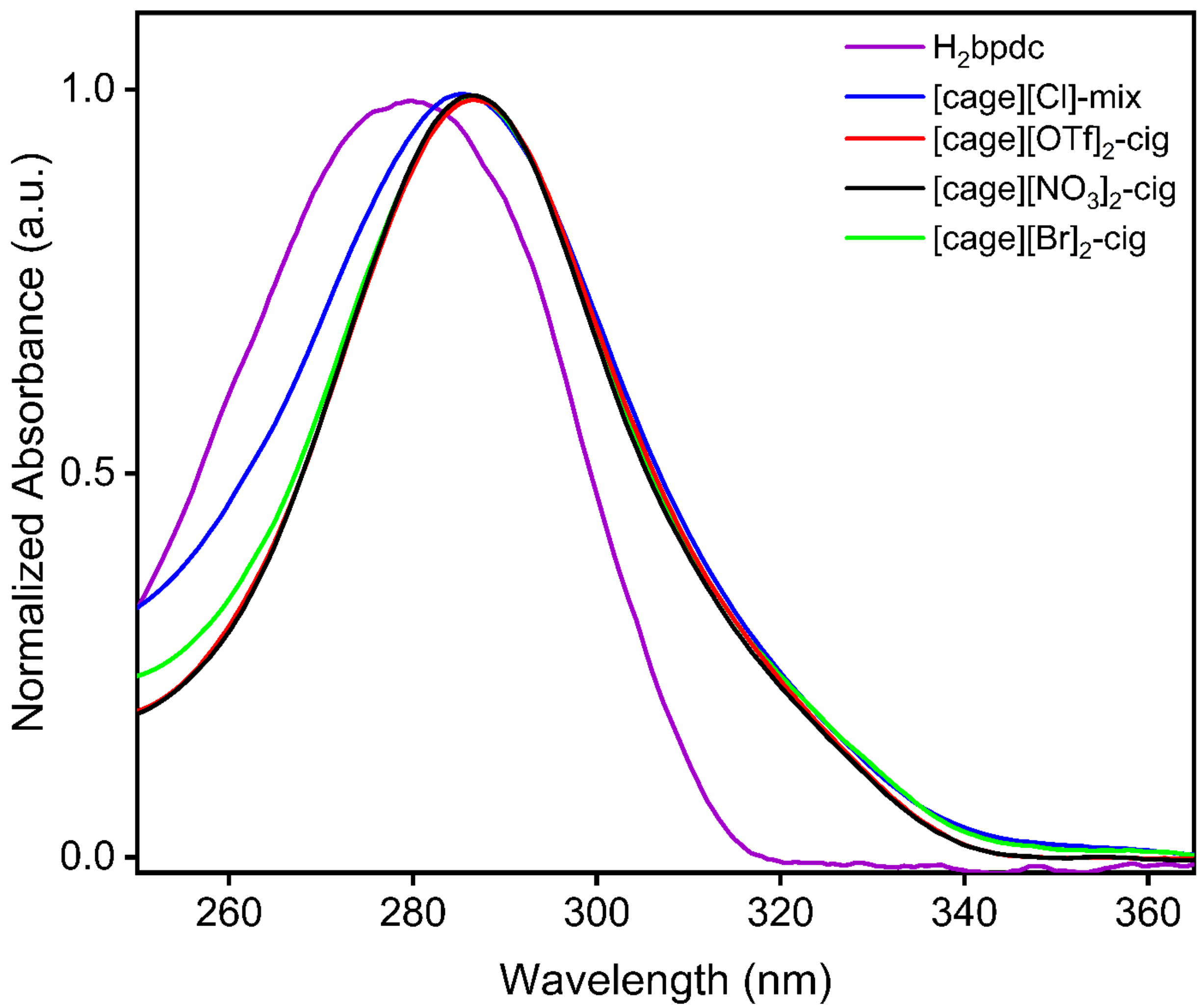

Figure S11. Normalized absorbance spectra of 3 equiv. H_2bpdc (purple), [cage][Cl]-mix (blue), [cage][OTf]$_2$-cig (red), [cage][NO$_3$]$_2$-cig (black), and [cage][Br]$_2$-cig (green) as 5 μM solutions in MeOH.

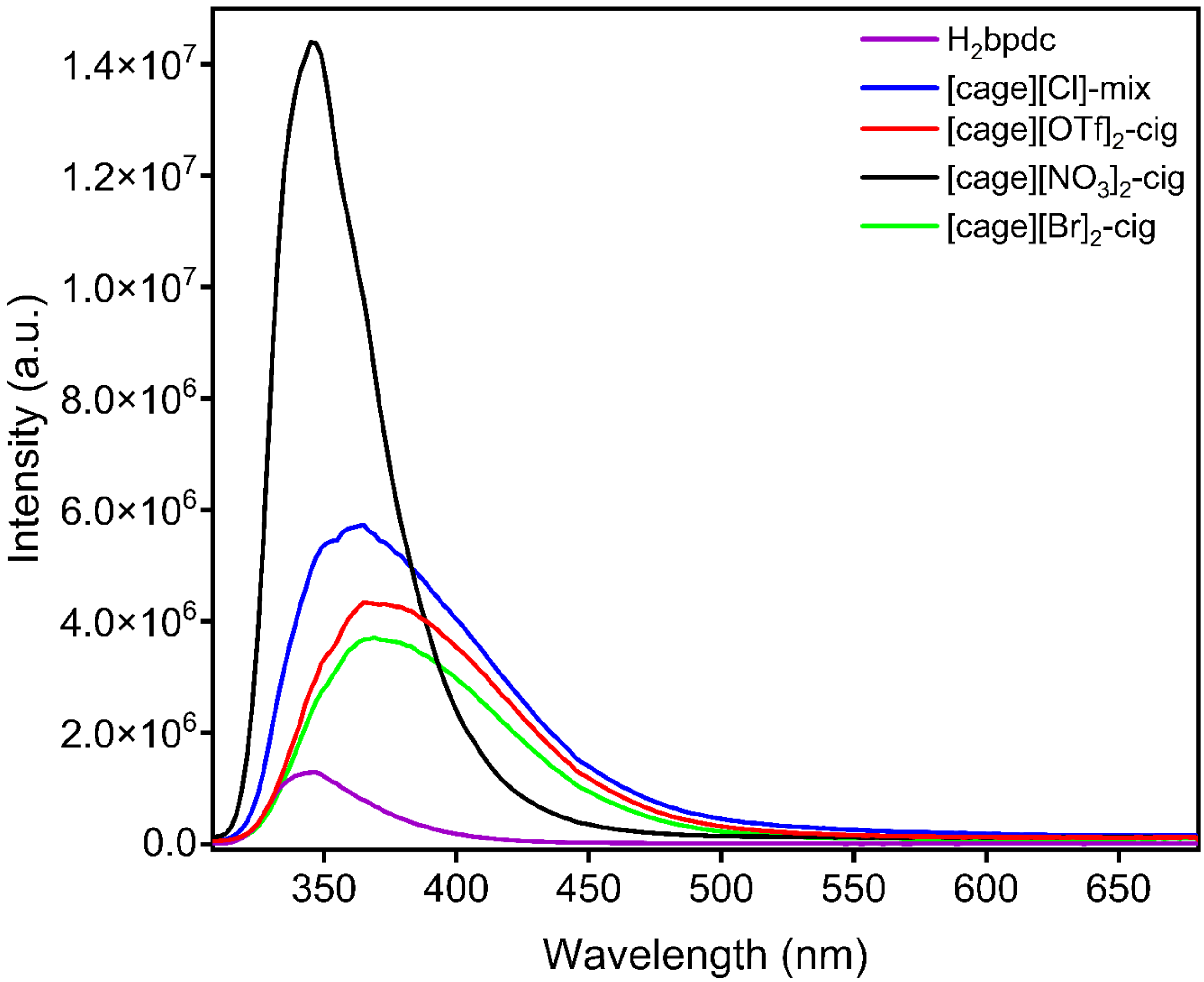

Figure S12. Solution fluorescence emission spectra of 3 equiv. H_2bpdc (purple), [cage][Cl]-mix (blue), [cage][OTf]$_2$-cig (red), [cage][NO_3]$_2$-cig (black), and [cage][Br]$_2$-cig (green) as 5 μM solutions in MeOH.

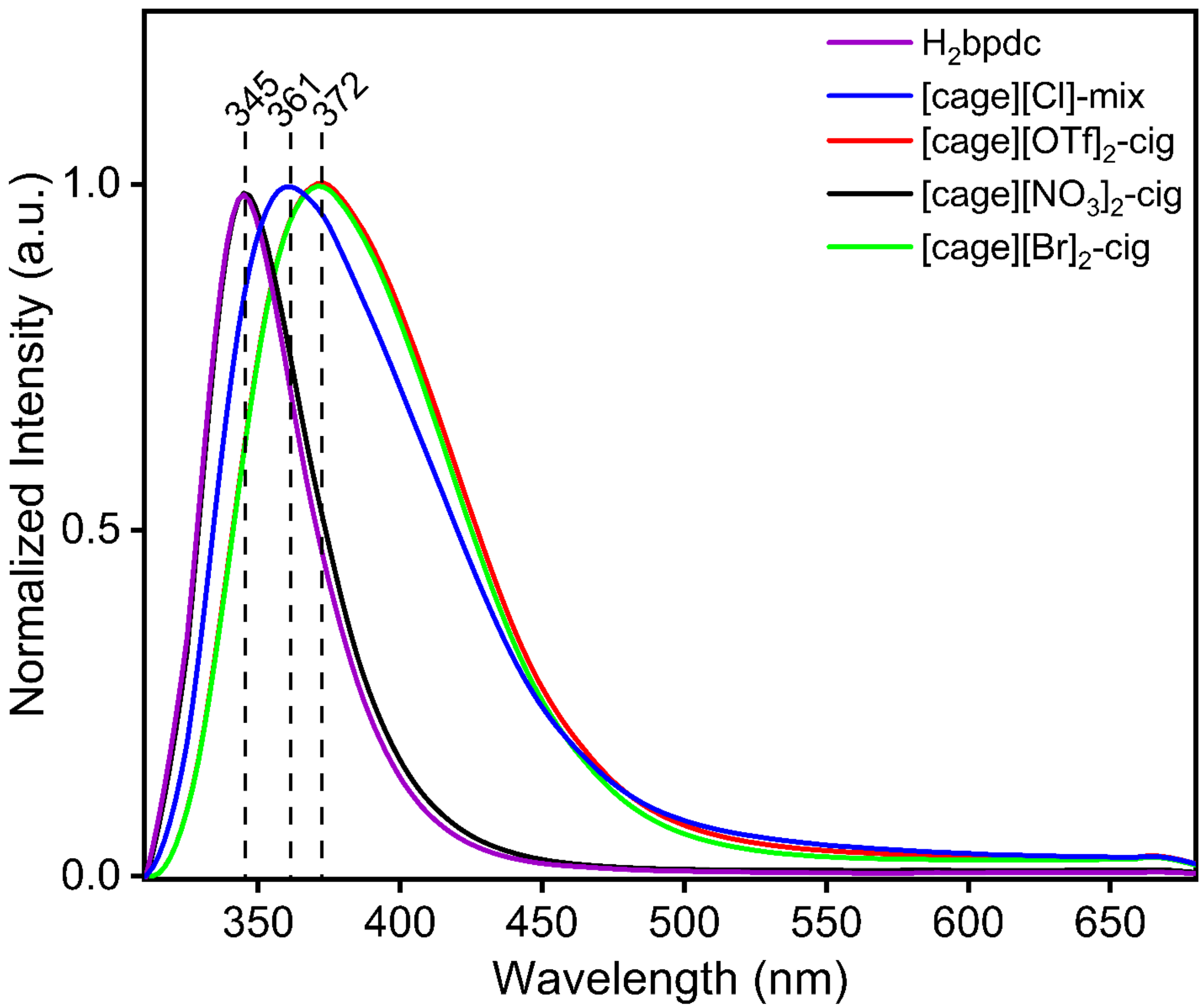

Figure S13. Normalized solution fluorescence emission spectra of 3 equiv. H_2bpdc (purple), [cage][Cl]-mix (blue), [cage][OTf]$_2$-cig (red), [cage][NO_3]$_2$-cig (black), and [cage][Br]$_2$-cig (green) as 5 μM solutions in MeOH.

Table S2. Peak positionings (nm) from absorbance and fluorescence data of H_2bpdc, [cage][Cl]-mix, [cage][OTf]$_2$-cig, [cage][NO_3]$_2$-cig, and [cage][Br]$_2$-cig as 5 μM solutions in MeOH.

Sample	Absorbance	Fluorescence
H_2bpdc	282	345
[cage][Cl]-mix	284	361
[cage][OTf]$_2$-cig	288	372
[cage][NO_3]$_2$-cig	286	345
[cage][Br]$_2$-cig	285	372

E. Fluorescence-based Anion Recognition Data

Fluorescence Emission Pre and Post Storage

Table S3. Mean max relative fluorescence unit (RFU) values of H_2bpdc, [cage][Cl]-mix, and [cage][OTf]$_2$-cig solutions (2600 μM) as triplicates. Data was collected with freshly made solutions (Initial) and one week later (After Storage).

Sample	Mean Max RFU – Initial	Mean Max RFU – After Storage
H_2bpdc	5923	12019
H_2bpdc	6098	11303
H_2bpdc	5923	10777
[cage][Cl]-mix	701	1198
[cage][Cl]-mix	690	1276
[cage][Cl]-mix	673	1310
[cage][OTf]$_2$-cig	363	406
[cage][OTf]$_2$-cig	375	395
[cage][OTf]$_2$-cig	365	399

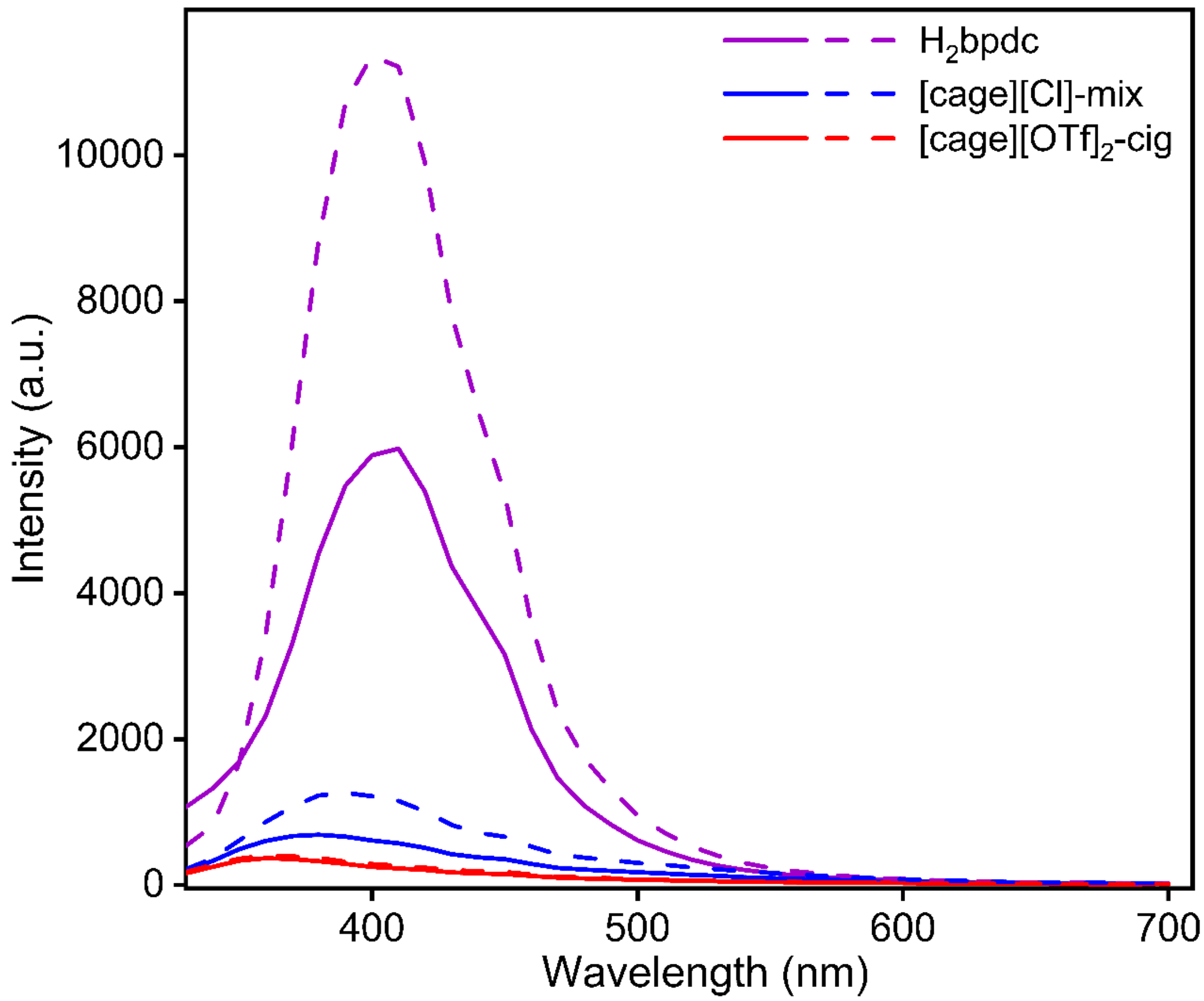

Figure S14. Solution state emission spectra of 2600 µM H_2bpdc (purple), [cage][Cl]-mix (blue), and [cage][OTf]$_2$-cig (red) in MeOH initially made (solid trace) and after storing for 1 week (dashed trace). There is an intensity increase observed for H_2bpdc and [cage][Cl]-mix because overtime more sample was able to go into solution.

Broad Salt Screening

Table S4. Resulting max fluorescence intensities of the 44 salts (10 eq.) that were introduced to 1 eq. [cage][OTf]$_2$-cig. The entries denoted as "[cage][OTf]$_2$-cig" are representative of the cells that only contained the starting triflate cage for calibration. Solvent was evaporated before data collection. Fluorescence emission intensity was normalized with absorbance data at excitation wavelength (λ_{ex} = 287 nm).

Salt added to [cage][OTf]$_2$-cig	Max Intensity	Salt added to [cage][OTf]$_2$-cig	Max Intensity
$Mg(NO_3)_2$	16725	LiCl	712
KNO_3	15405	$NiCl_2$	693
$Ni(NO_3)_2$	13924	$CoCl_2$	684
$Al(NO_3)_3$	13810	CoF_2	655
$Cu(NO_3)_2$	13454	$NaBO_3$	620
$Co(NO_3)_2$	13274	$NaNO_2$	596
$Cr(NO_3)_3$	10339	KF	531
NaBr	8484	NH_4PF_6	507
KBr	6247	FeF_3	471
$C_{19}H_{42}NBr$	2925	$NaHSO_3$	450
TBABr	2281	NH_4F	410
$(NH_4)_2S_2O_8$	1404	$NaIO_4$	382
Na_2SO_4	1186	$NaCF_3CO_2$	369
$BaSO_4$	1075	$CuSO_4$	365
$Co(BF_4)_2$	1008	NaI	361
TBAI	915	CoF_3	214
$CuCO_3$	894	NaSCN	192
FeF_2	874	$Fe_2(SO_4)_3$	188
$MnCl_2$	842	TBAF	177
TMACl	828	$KMnO_4$	171
KI	806	[cage][OTf]$_2$-cig	735
$MgCl_2$	779	[cage][OTf]$_2$-cig	717
CuBr	740	[cage][OTf]$_2$-cig	560
K_2CO_3	728		

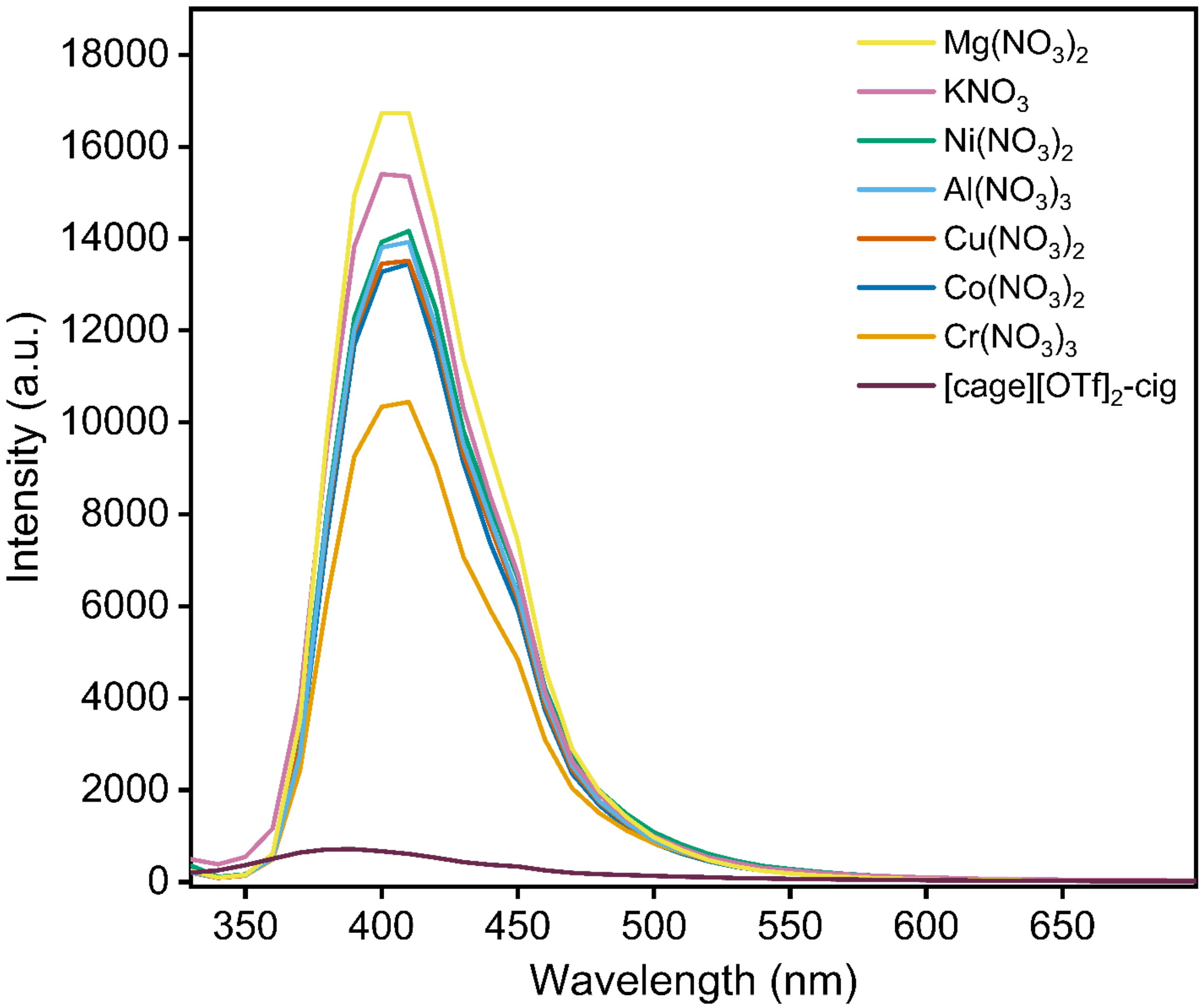

Figure S15. Normalized emission spectra of [cage][OTf]$_2$-cig and its anion exchange with varying nitrate salts. The addition of 10 equivalents nitrate salts to 1 equivalent cage enhances fluorescence, where $Mg(NO_3)_2$ results in the brightest emission. Solvent was evaporated before data collection.

Concentration Dependence of $Mg(NO_3)_2 \cdot 6H_2O$

Table S5. Relationship between $Mg(NO_3)_2$ concentration introduced, the equivalence of $Mg(NO_3)_2$ to [cage][OTf]$_2$-cig, and the resulting max fluorescence intensities. The bottom three rows are representative of the cells that only contained [cage][OTf]$_2$-cig for calibration. Solvent was evaporated before data collection. Fluorescence emission intensity was normalized with absorbance data at excitation wavelength (λ_{ex} = 287 nm).

Added $Mg(NO_3)_2$ (μM)	$Mg(NO_3)_2$ Equivalents	Max Intensity
100,000	12.5	39086
95,000	11.875	63960
90,000	11.25	19212
85,000	10.625	22245
80,000	10	24800
75,000	9.375	19039
70,000	8.75	21259
65,000	8.125	16435
60,000	7.5	30556
55,000	6.875	11461
50,000	6.25	12541
45,000	5.625	20422
40,000	5	12700
35,000	4.375	11091
30,000	3.75	12573
25,000	3.125	13807
20,000	2.5	23241
15,000	1.875	20924
10,000	1.25	38094
5,000	0.625	26987
1,000	0.125	8481
750	0.09	3252
250	0.03	40
0	0	816
0	0	936
0	0	552

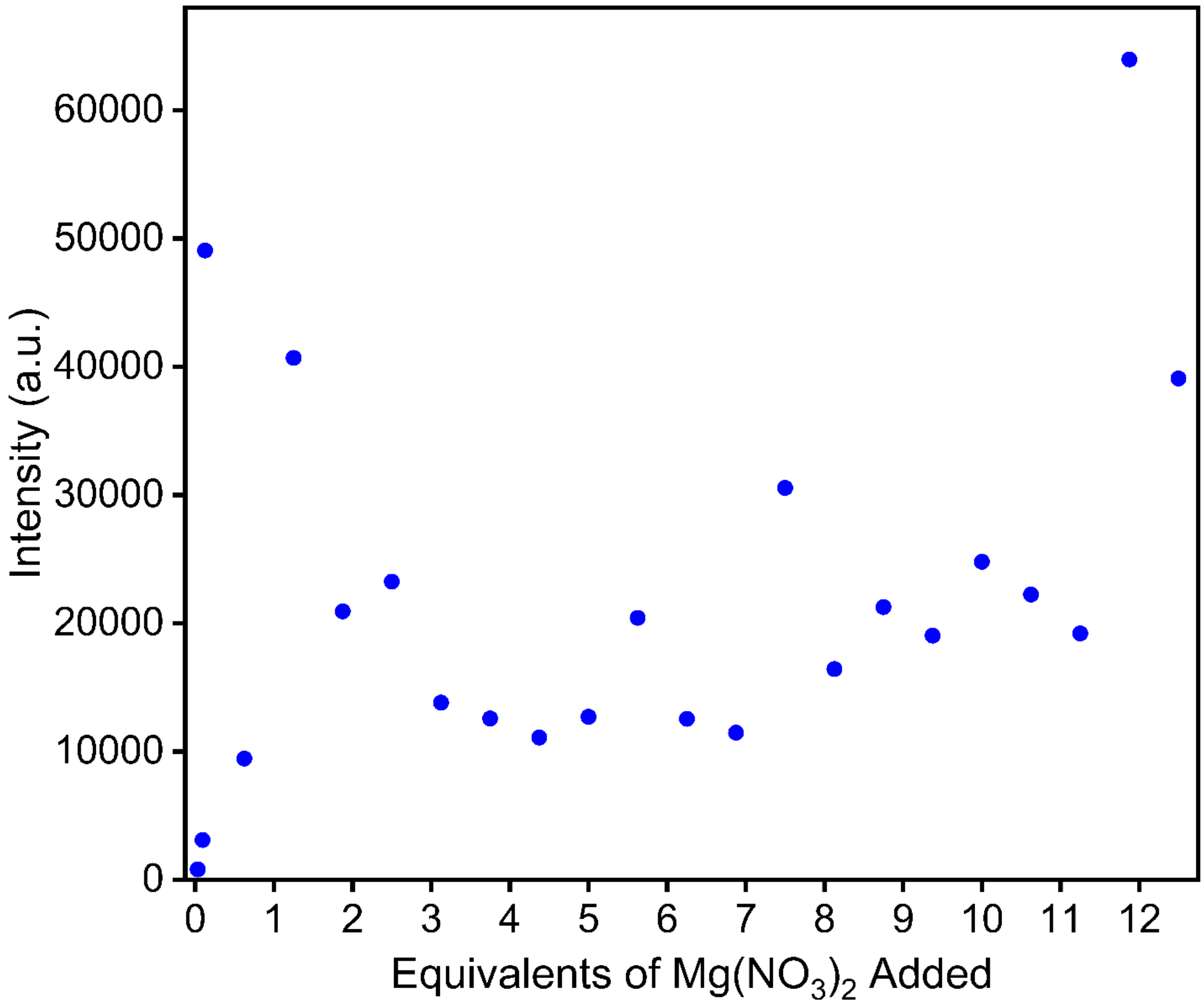

Figure S16. Max intensities of $Mg(NO_3)_2$ equivalent to one equivalent of [cage][OTf]$_2$-cig. Fluorescence emission intensity was normalized with absorbance data at excitation wavelength (λ_{ex} = 287 nm).

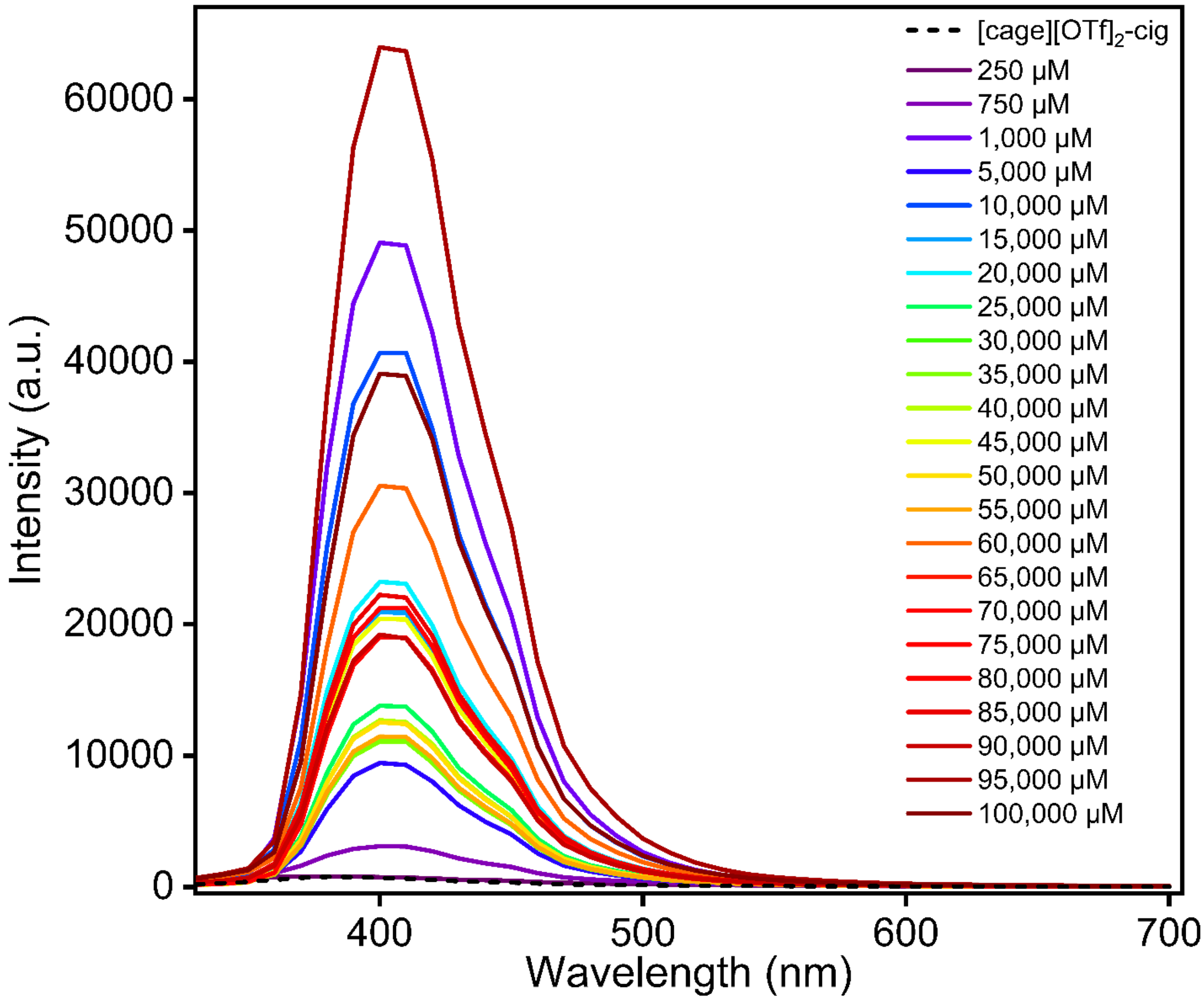

Figure S17. Emission spectra of averaged [cage][OTf]$_2$-cig and its resulting fluorescence from the introduction to various concentrations of $Mg(NO_3)_2$ solutions. Solvent was evaporated before data collection. Fluorescence emission intensity was normalized with absorbance data at excitation wavelength (λ_{ex} = 287 nm).

F. Single crystal X-ray diffraction (SCXRD)

$[Zr_6(\mu_3\text{-}O)_2(\mu_2\text{-}OH)_6(Cp)_6(bpdc)_3][NO_3]_2$ ($[cage][NO_3]_2$-cig):

Needle-shaped crystal was placed on a Kapton mount with inert oil for crystal structure determination. The data collection was carried out using a Bruker D8 Venture KAPPA diffractometer equipped with a microfocus sealed tube and Mo-Kα radiation (λ = 0.71073 Å) and a multilayer mirror monochromator. The experiment was performed at 153(2) K using an Oxford Cryostream open-nitrogen-flow temperature control unit.

Unit cell parameters were obtained using 1.0° ω and φ scans, 15 s frame time, and a detector distance of 60 mm. The total exposure time was 4.3 hours. The frames were integrated with the Bruker SAINT software package using a narrow-frame algorithm.[2] The integrations of the data using a monoclinic unit cell yielded 169602 reflections to a maximum θ angle of 26.40° (0.80 Å resolution), of which 14783 were independent (average redundancy 11.47, completeness = 99.9%, R_{int} = 7.01%, R_{sig} = 3.50%) and 10142 (68.6%) were greater than $2\sigma(F^2)$. The final cell constants of a = 21.2452(9) Å, b = 15.9854(7) Å, c = 21.8297(9) Å, β = 102.9120(10)°, volume = 7226.2(5) $Å^3$, are based upon the refinement of the XYZ-centroids of 9933 reflections above 20 $\sigma(I)$ with 2.30° < 2θ < 26.31°. Data were corrected for absorption effects using the Multi-Scan method in SADABS 2016/2. The calculated minimum and maximum transmission coefficients (based on crystal size) are 0.882 and 0.973.[3] Please refer to Table S4 for additional crystal and refinement information.

The space group $P2_1/n$ was determined based on intensity statistics and systematic absences. The structure was solved by SHELXT 2018.2 and refined with full-matrix least squares / difference Fourier cycles using SHELXL-2019/2; Z = 4 for the formula unit $C_{72}H_{60}N_2O_{26}Zr_6$+[solvent].[3,4] Non-hydrogen atoms were refined with anisotropic displacement parameters. The hydrogen atoms were placed in ideal positions and refined as riding atoms with relative isotropic displacement parameters. The final anisotropic full-matrix least-squares refinement on F^2 with 1074 variables against 14783 data points and 599 restraints converged at R_1 = 5.28%, for the observed data and wR_2 = 15.27% for all data. The goodness-of-fit on F^2 was 1.04. The largest peak in the final difference electron density synthesis was 1.57 $e^-/Å^3$ and the deepest hole was -0.94 $e^-/Å^3$ with an RMS deviation of 0.108 $e^-/Å^3$. On the basis of the final model, the calculated density was 1.76 g/cm^3 and $F(000)$, 3816 e^-.

The reflections 0 0 2, 2 0 0, 1 1 1, and 0 1 2 were omitted due to suspected interference from the beamstop. There was a small amount of unresolved solvent present in the sample. Attempts to model this solvent as MeOH were unsuccessful, and the SQUEEZE function of PLATON was used to remove the electron density associated with the solvent.[6] SQUEEZE found 57 electrons in 298 $Å^3$ of solvent accessible volume per unit cell.

The overall packing of the structure consists of "columns" of the cluster linked by hydrogen bonding to the nitrate anions. These columns are oriented parallel to the c-axis of the unit cell. Each cluster has hydrogen bond interactions on either end of the cluster between the hydroxide linkers and the nitrate anion. There are three hydroxide linkers on either end of the cluster and only two nitrate anions per cluster, and this causes disorder of the nitrate anions. One of the anions is in a position where it is fully occupied and forming a hydrogen bond to the hydroxide group consisting of O2 and H2O, while the other nitrate anion is disordered as partially occupied over two locations. The approximate occupancy ratio is 66:34. In the majorly occupied position, the nitrate is forming a hydrogen bond with the hydroxide group consisting of O18 and H18O. In the minorly occupied, the anion is forming a hydrogen bond with the hydroxyl group consisting of O19 and H19O.

Two of the Cp ligands are rotationally disordered and modeled in two partially occupied parts. For the Cp consisting of carbons 1–5 and carbons 1'–5', the approximate occupancy ratio is 45:55. For the Cp consisting of carbons 58–62 and carbons 58'–62', the approximate occupancy ratio is 59:41.

For the biphenyl linkers, one end of each of the linkers is rotationally disordered and modeled in two partially occupied parts. The occupancy ratio for the disordered parts of each linker is correlated, and the approximate occupancy ratio is 89:11.

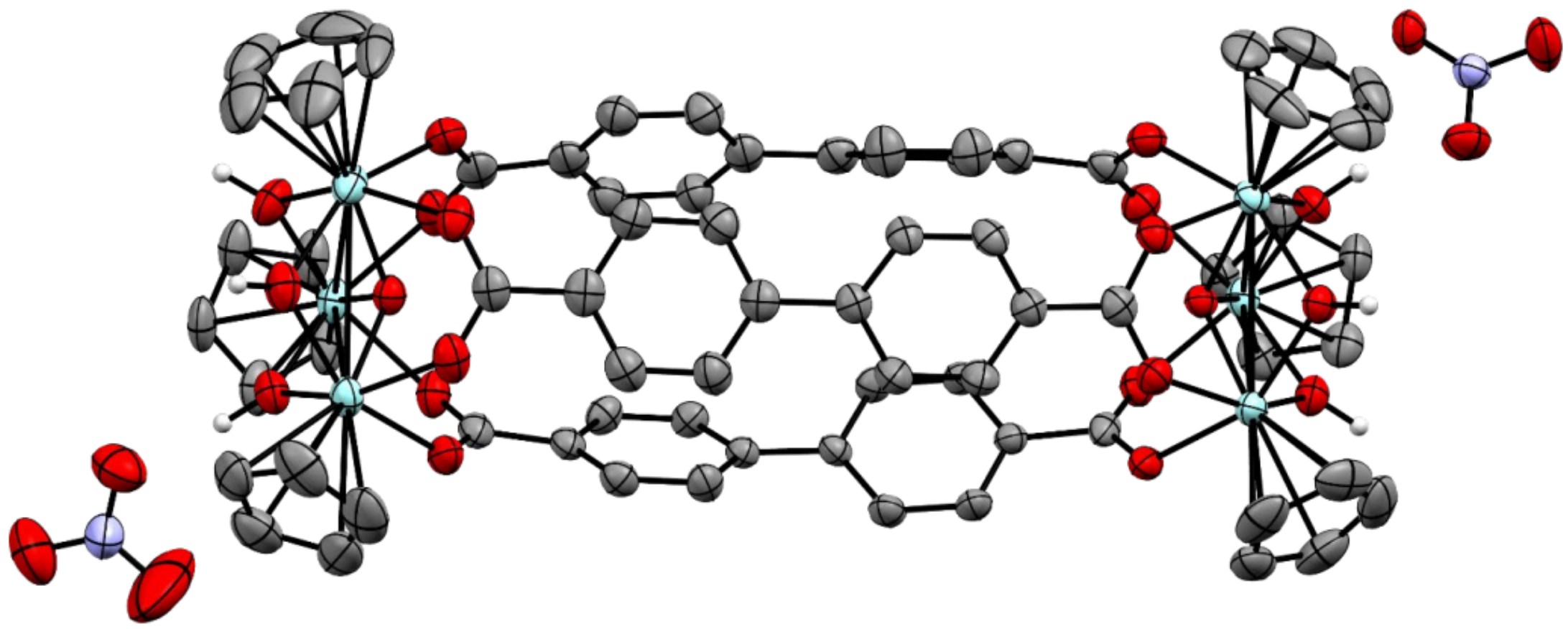

Figure S18. Crystal structure of [cage][NO_3]$_2$-cig. Most hydrogen atoms have been omitted for clarity. Carbon, oxygen, zirconium, and nitrogen atoms are represented as grey, red, teal, and purple thermal ellipsoids at the 50% probability level, respectively. Hydrogen atoms are drawn as fixed size spheres.

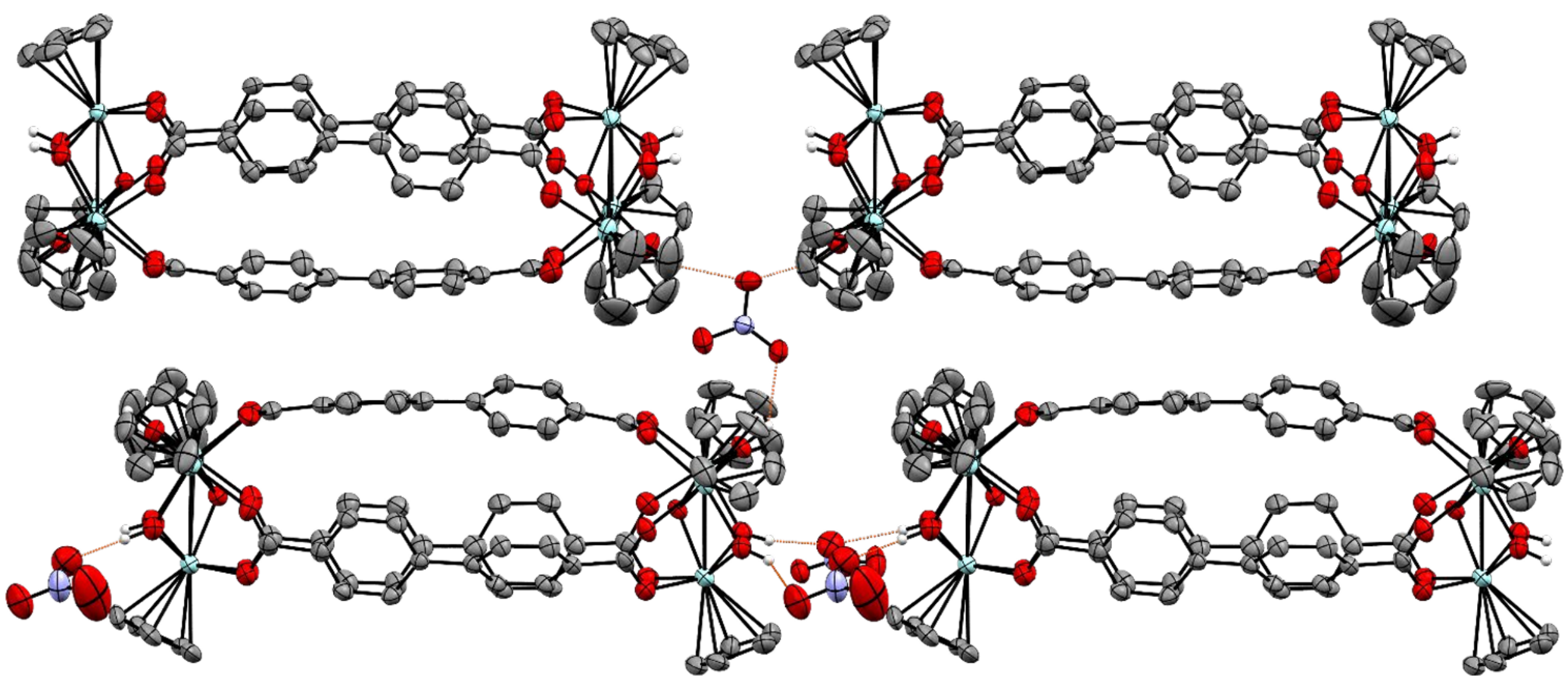

Figure S19. Hydrogen bonding between the clusters and the nitrate anions. Only the majorly occupied parts of the cluster are shown. Most of the hydrogen atoms are omitted for clarity.

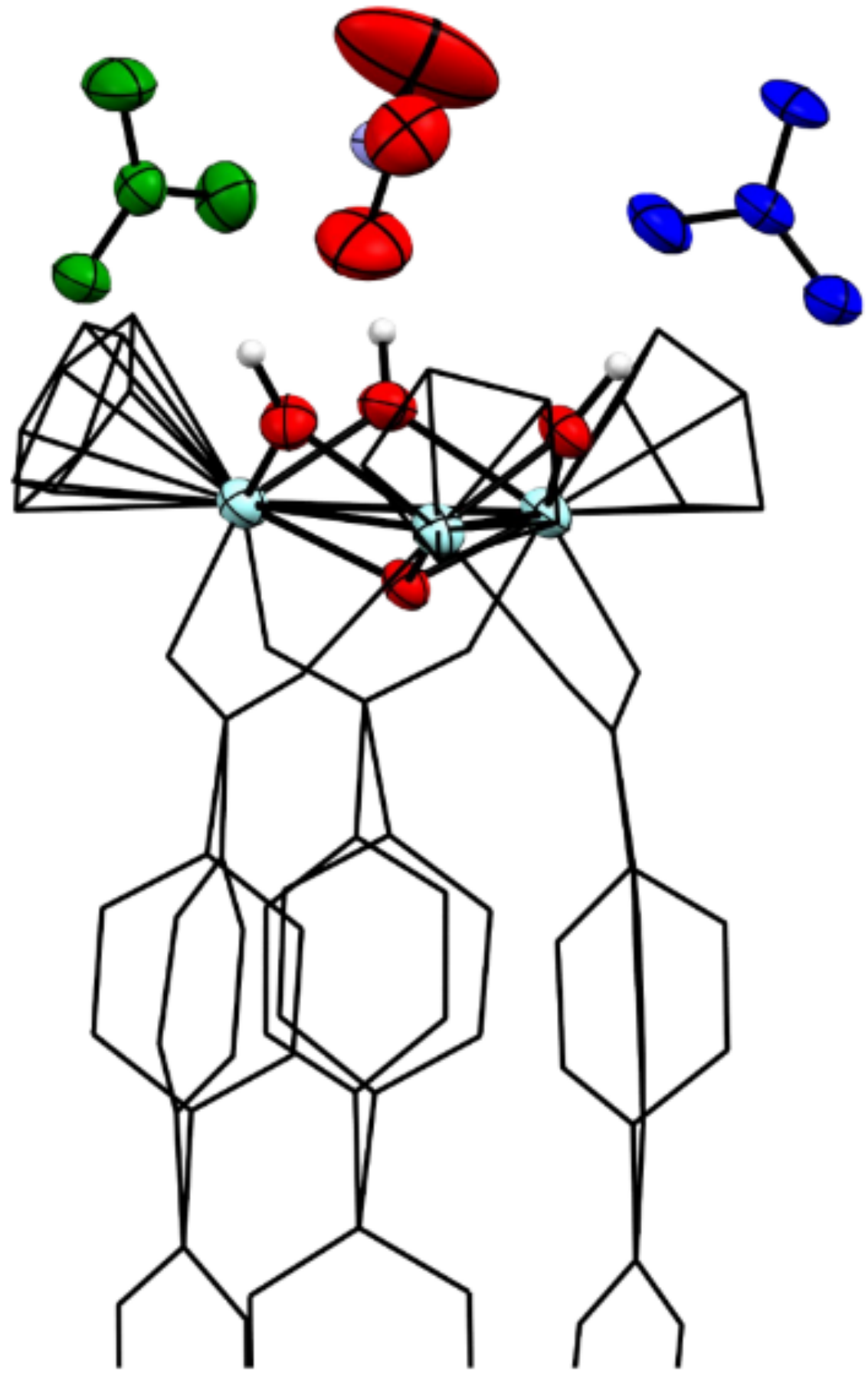

Figure S20. The three locations of the nitrate anions. The anions colored green and blue are partially occupied, with the green anion having an approximate occupancy of 66% and the blue anion having an approximate occupancy of 34%. Most of the cluster has been drawn in wireframe and most of the hydrogen atoms have been omitted for clarity.

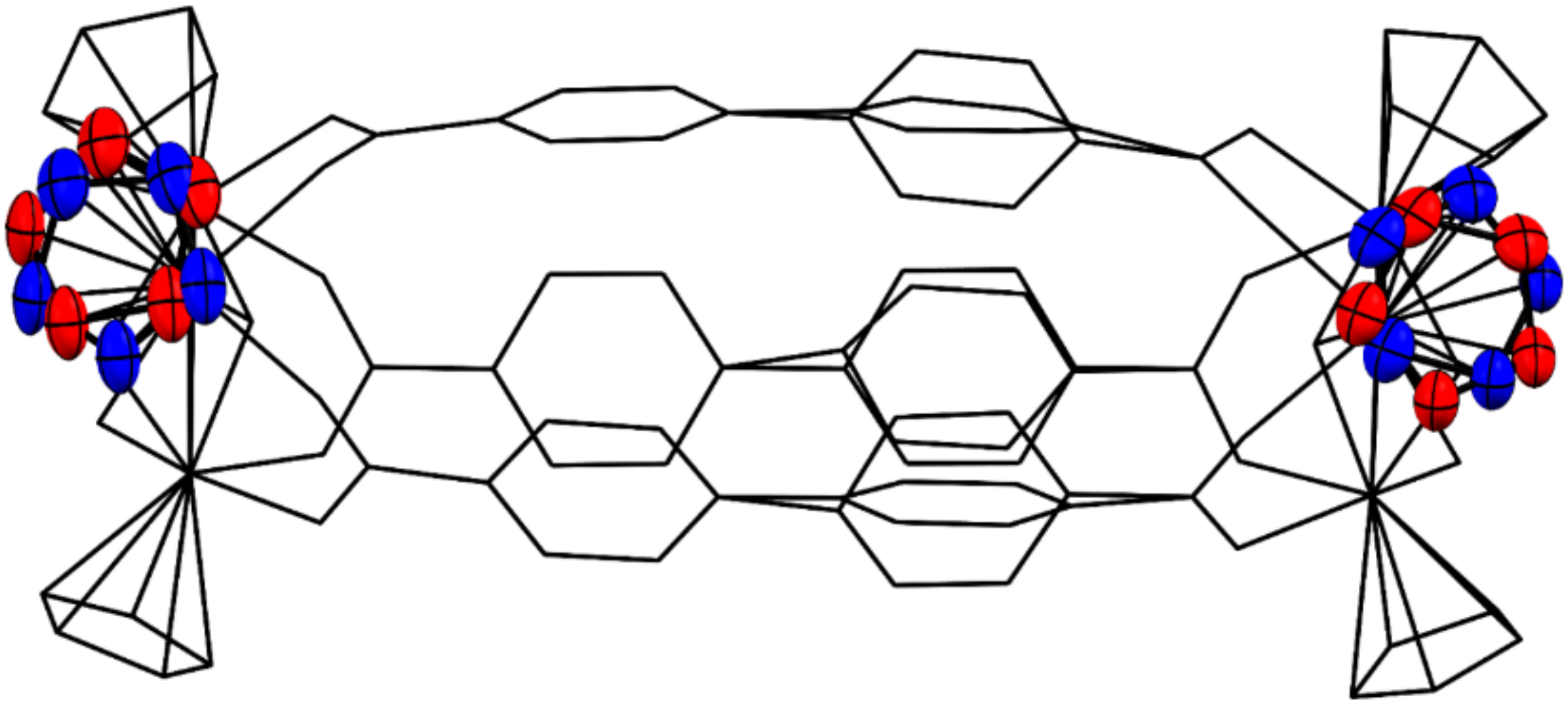

Figure S21. The disorder of the Cp ligands. Each disordered part is colored red or blue. Most of the cluster has been drawn in wireframe and hydrogen atoms have been omitted for clarity.

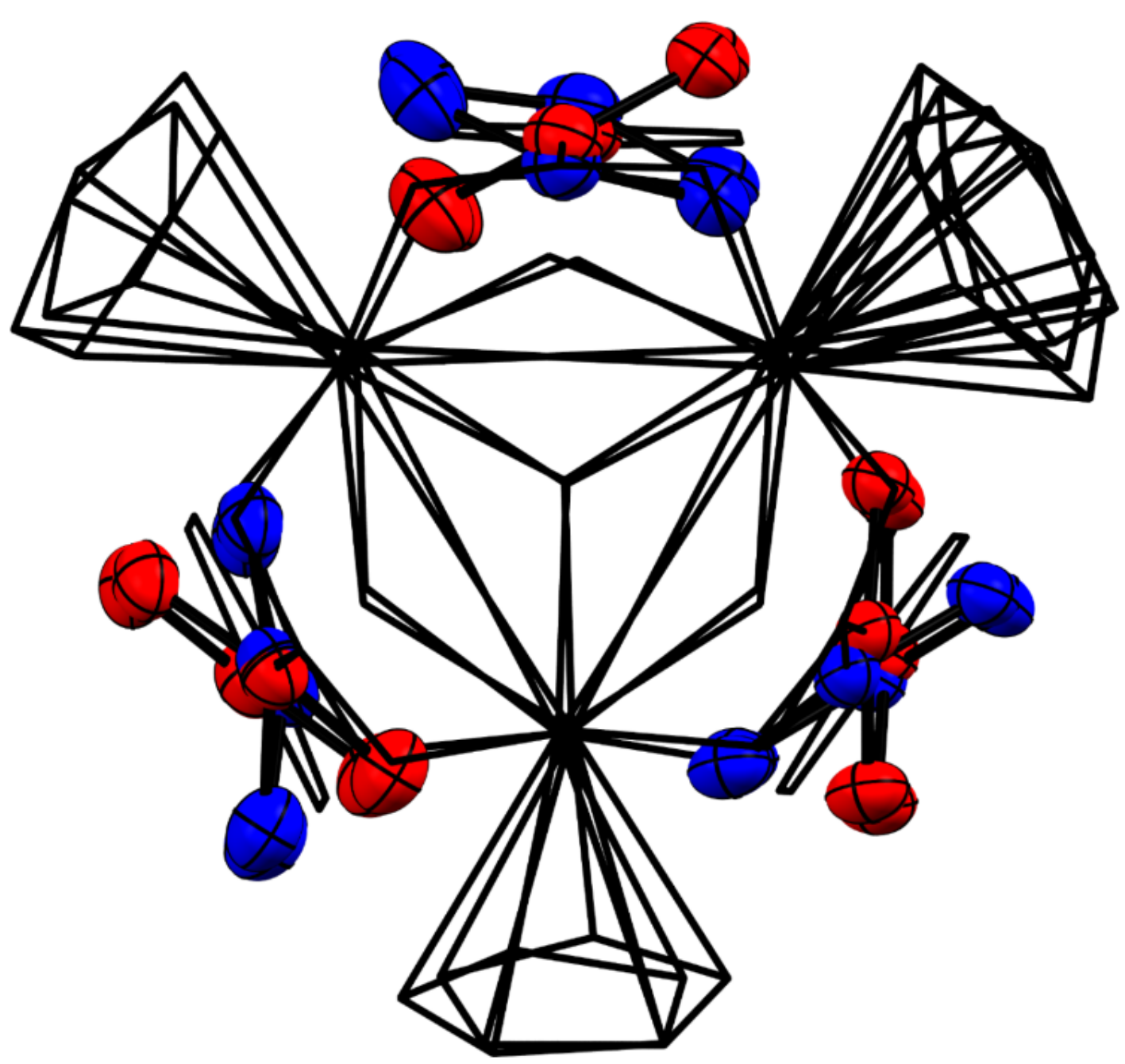

Figure S22. The disorder in the biphenyl linkers. Each part colored red and blue have equal occupancies, with the red position being majorly occupied. Most of the cluster has been drawn in wireframe and hydrogen atoms have been omitted for clarity.

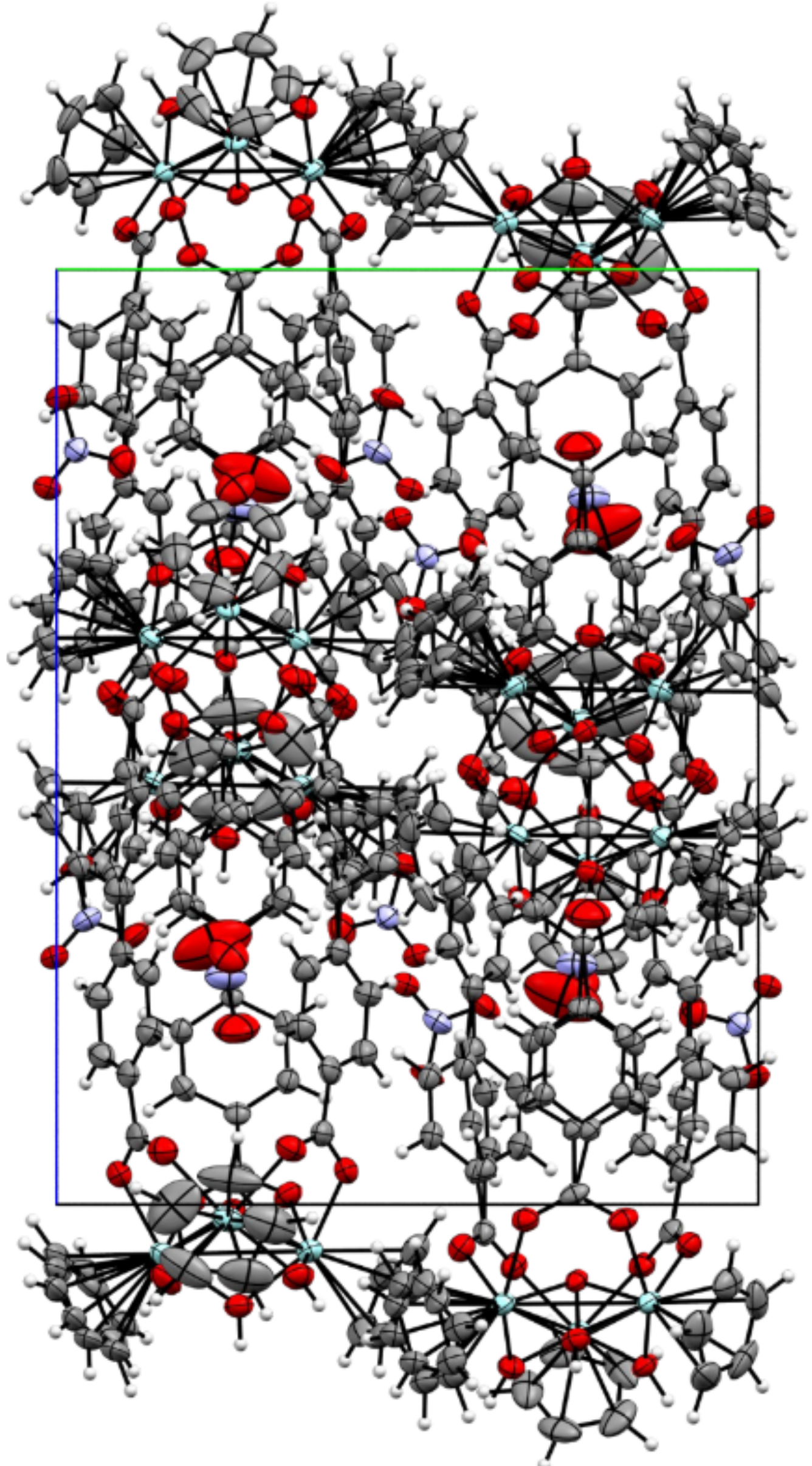

Figure S23. A packed unit cell of [cage][NO_3]$_2$-cig viewed along the crystallographic a-axis. The green and blue axes are the b- and c-axis respectively. All disordered parts are shown.

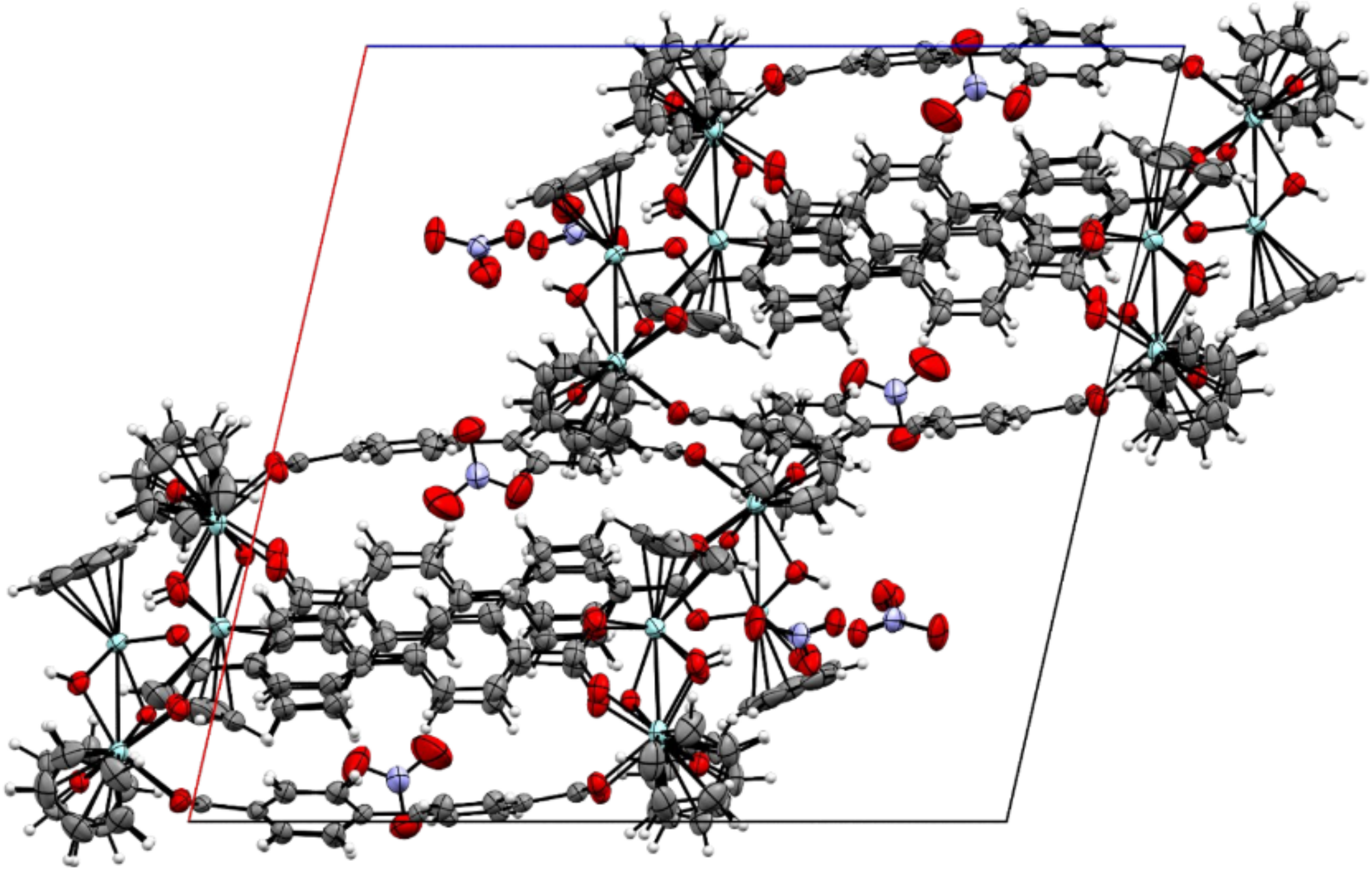

Figure S24. A packed unit cell of [cage][NO_3]$_2$-cig viewed along the crystallographic b-axis. The red and blue axes are the a- and c-axis respectively. All disordered parts are shown.

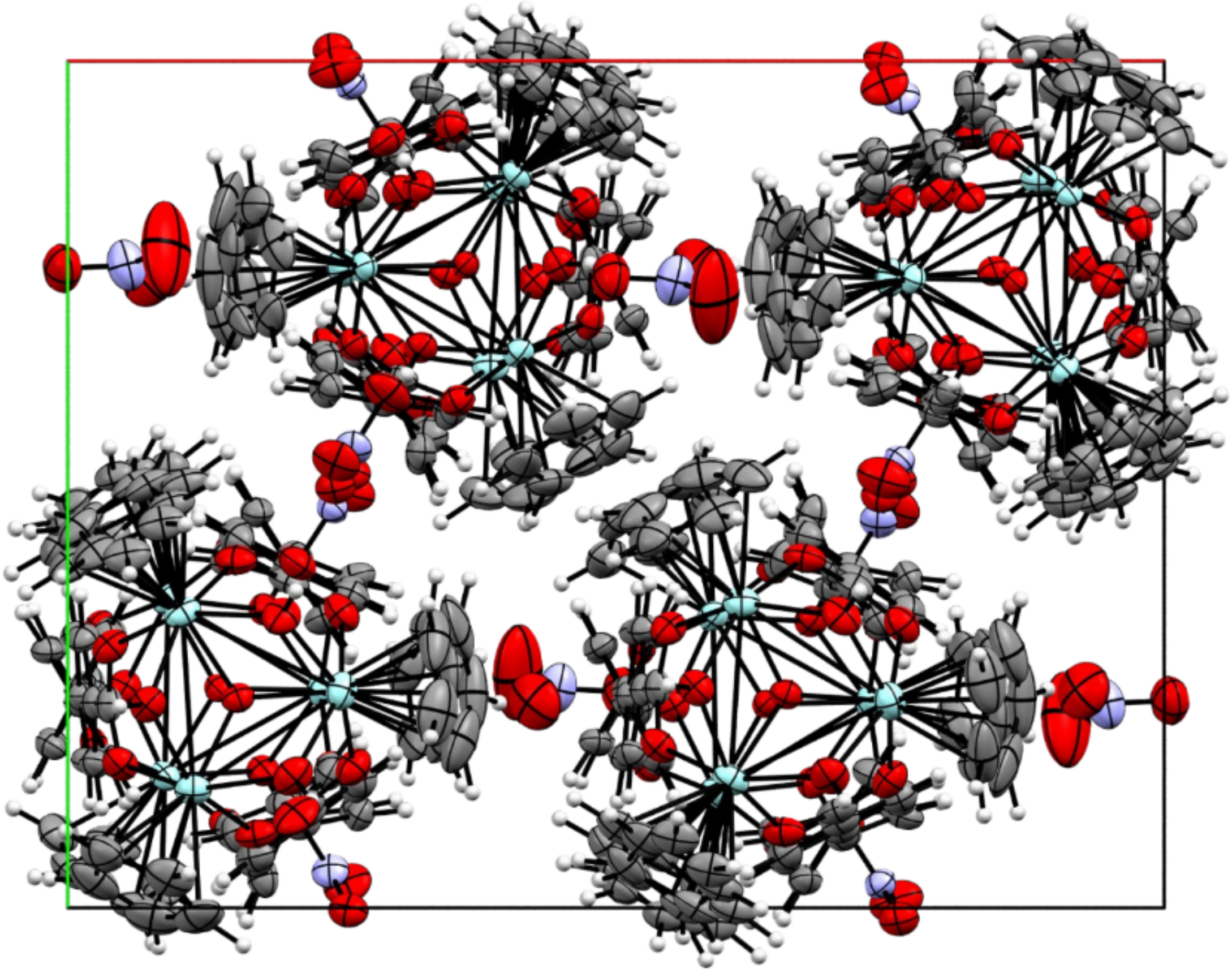

Figure S25. A packed unit cell of [cage][NO_3]$_2$-cig viewed along the crystallographic c-axis. The red and green axes are the a- and b-axis respectively. All disordered parts are shown.

Table S6. SCXRD crystal data and structure refinement for [cage][NO_3]$_2$-cig.

Empirical formula	$C_{72}H_{60}N_2O_{26}Zr_6$+[solvent]
Formula weight	1916.54
Crystal color, shape, size	Colorless needle, 0.03 × 0.04 × 0.14 mm^3
Temperature	153(2) K
Crystal system, space group	Monoclinic, $P2_1/n$
Unit cell dimensions	a = 21.2452(9) Å α = 90°<br>b = 15.9854(7) Å β = 102.9120(10)°<br>c = 21.8297(9) Å ɣ = 90°
Volume	7226.2(5) $Å^3$
Z	4
Density (calculated)	1.762 g/cm^3
Absorption coefficient	0.917 mm^{-1}
F(000)	3816
Radiation	MoK_α (λ = 0.71073 Å)
2θ range	4.65° to 52.80° (0.80 Å)
Index ranges	-25 ≤ h ≤ 26<br>-19 ≤ k ≤ 19<br>-27 ≤ l ≤ 27
Reflections collected	169602
Independent reflections	14783<br>R_{int} = 0.0701<br>R_{sigma} = 0.0350
Completeness to θ = 25.242°	99.9
Data / Restraints / Parameters	14783 / 599 / 1074
Absorption correction Tmin/Tmax	0.882 / 0.973 (Multi-Scan method)
Goodness-of-fit on F^2	1.045
Final *R* indexes [$I \geq 2\sigma(I)$]	R_1 = 0.0528<br>wR_2 = 0.1262
Final *R* indexes [all data]	R_1 = 0.0867<br>wR_2 = 0.1527
Largest peak/hole	1.57/-0.94 e$Å^{-3}$

$[Zr_6(\mu_3\text{-}O)_2(\mu_2\text{-}OH)_6(Cp)_6(bpdc)_3][Br]_2$ ([cage][Br]$_2$-cig):

A colorless, block shaped crystal was placed on a MiTeGen mount with inert oil for crystal structure determination. The data collection was carried out using a Bruker D8 VENTURE diffractometer equipped with a microfocus sealed tube (λ = 0.71073 Å) and a multilayer mirror monochromator. The specimen was held at 153(2) K during the measurement with a Cryostream low temperature device.

Unit cell parameters were obtained using 1.0° ω and φ scans, frame times of 1 s, and a detector distance of 80 mm, with a total exposure time of 2.5 hours. The frames were integrated with the SAINT V8.41 package.[2] The integration of the data using a monoclinic unit cell yielded a total of 178490 reflections to a maximum θ angle of 30.55° (0.70 Å resolution), of which 21890 were independent (average redundancy 8.15, completeness = 99.9%, R_{int} = 6.86%, R_{sig} = 4.08%) and 16197 (74.0%) were greater than $2\sigma(F^2)$. The final cell constants of a = 21.1656(7) Å, b = 16.0251(6) Å, c = 21.6374(7) Å, volume = 7151.0(4) Å^3, are based upon the refinement of the XYZ-centroids of 2096 reflections above 20 $\sigma(I)$ with 1.81° < 2θ < 32.05°. Data were corrected for absorption effects using the Multi-Scan method in SADABS 2016/2. The calculated minimum and maximum transmission coefficients (based on crystal size) are 0.641 and 0.750.[3] Please refer to Table S5 for additional crystal and refinement information.

The space group $P2_1/n$ (#14) was determined based on intensity statistics and systematic absences. The structure was solved by SHELXT 2018/2 and refined with full-matrix least squares / difference Fourier cycles using SHELXL-2019/2; Z = 4 for the formula unit $C_{73}H_{66}Br_2O_{22}Zr_6$.[4,5] The final anisotropic full-matrix least-squares refinement on F^2 with 961 variables against 21890 data points 1559 and 1559 restraints converged at R_1 = 3.57%, for the observed data and wR_2 = 9.31% for all data. The goodness-of-fit on F^2 was 1.03. The largest peak in the final difference electron density synthesis was 1.05 e$^-$/Å^3 and the deepest hole was −1.06 e$^-$/Å^3 with an RMS deviation of 0.102 e$^-$/Å^3. On the basis of the final model, the calculated density was 1.86 g/cm^3 and $F(000)$, 3960 e$^-$.

The reflection 0 0 2 was omitted due to suspected interference from the beamstop. There was a small amount of solvent present in the sample that was resolved as methanol and water.

The overall packing of the structure consists of "columns" of the cluster linked by hydrogen bonding to the bromide anions and solvent molecules. These columns are oriented parallel to the c-axis of the unit cell. There are three hydroxide linkers on either end of the cluster and only two bromide anions per cluster. Each cluster has hydrogen bond interactions on either end of the cluster with the hydroxide linkers. On one end, a hydroxide linker is hydrogen bonding to a bromide anion, which is in turn forming a hydrogen bond with the cocrystallized methanol. On the other end of the cluster, a hydroxide linker is hydrogen bonded to a cocrystallized water molecule, which is then

interacting with the second bromide anion of that cluster. This bromide anion is hydrogen bonding to a hydroxide linker on an adjacent cluster. There is disorder in the positions of the water and bromide anions, which show "swapped" positions in the crystal (Figure S26). The approximate occupancy ratio of the disordered positions is 84:16.

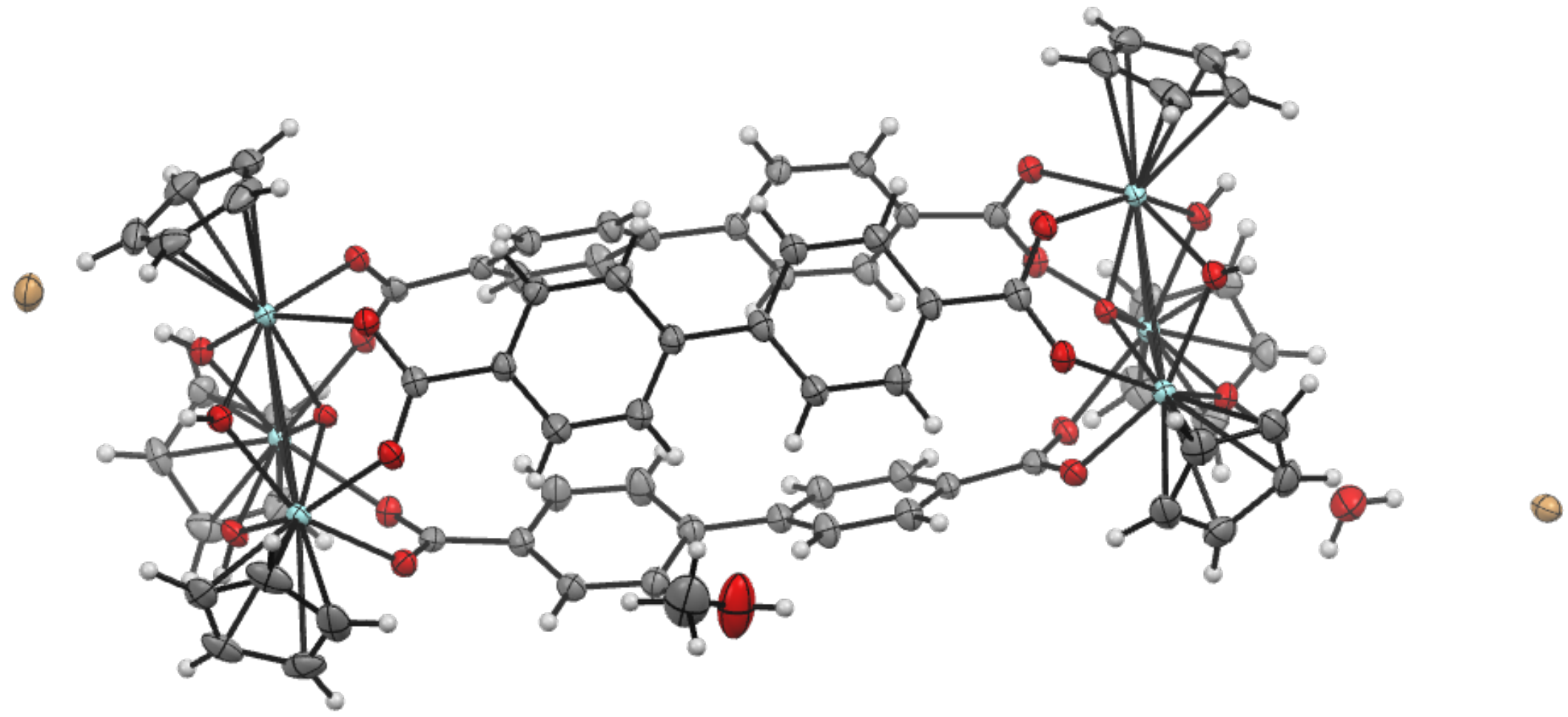

Figure S26. Crystal structure of [cage][Br]$_2$-cig. Only the majorly occupied parts are shown. Carbon, oxygen, zirconium, and bromine atoms are represented as grey, red, teal, and tan thermal ellipsoids at the 50% probability level. Hydrogen atoms are drawn as fixed size spheres.

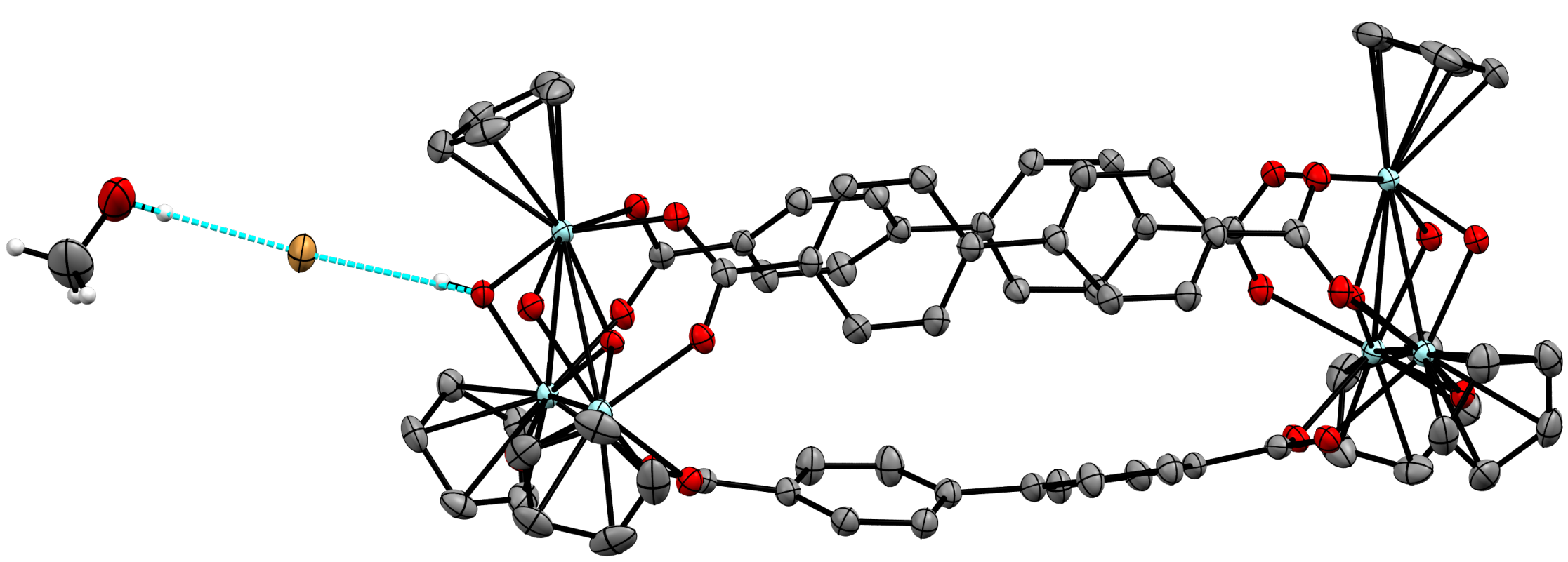

Figure S27. Hydrogen bonding between the cluster, a bromide anion, and the cocrystallized methanol. Hydrogen bonding interactions are shown as dotted lines. Most of the hydrogen atoms are omitted for clarity.

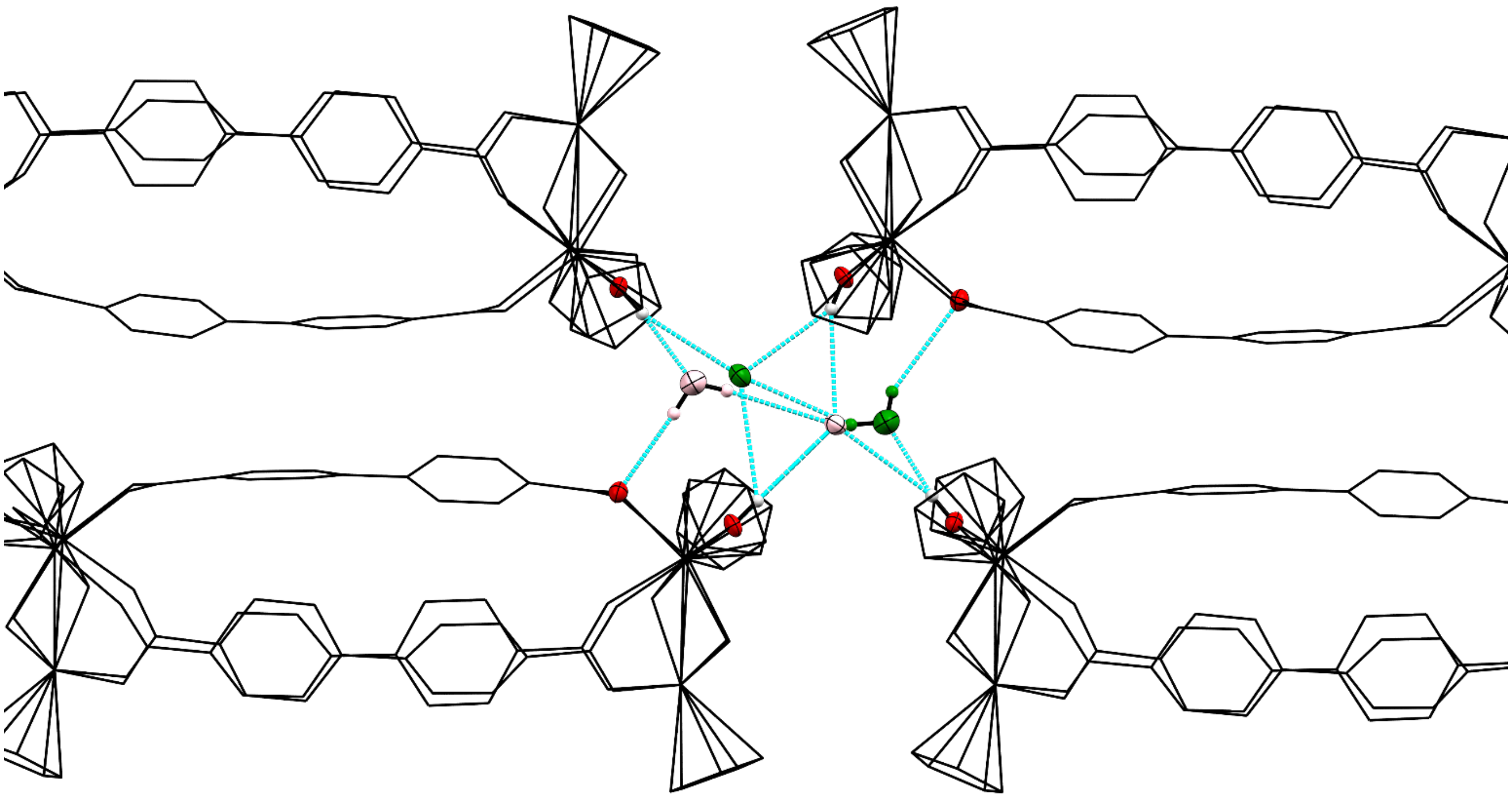

Figure S28. Disorder of the bromide anion and water molecule. The two disordered parts are colored pink and green for clarity. The pink position is the majorly occupied position. Hydrogen bonding interactions are shown as dotted lines.

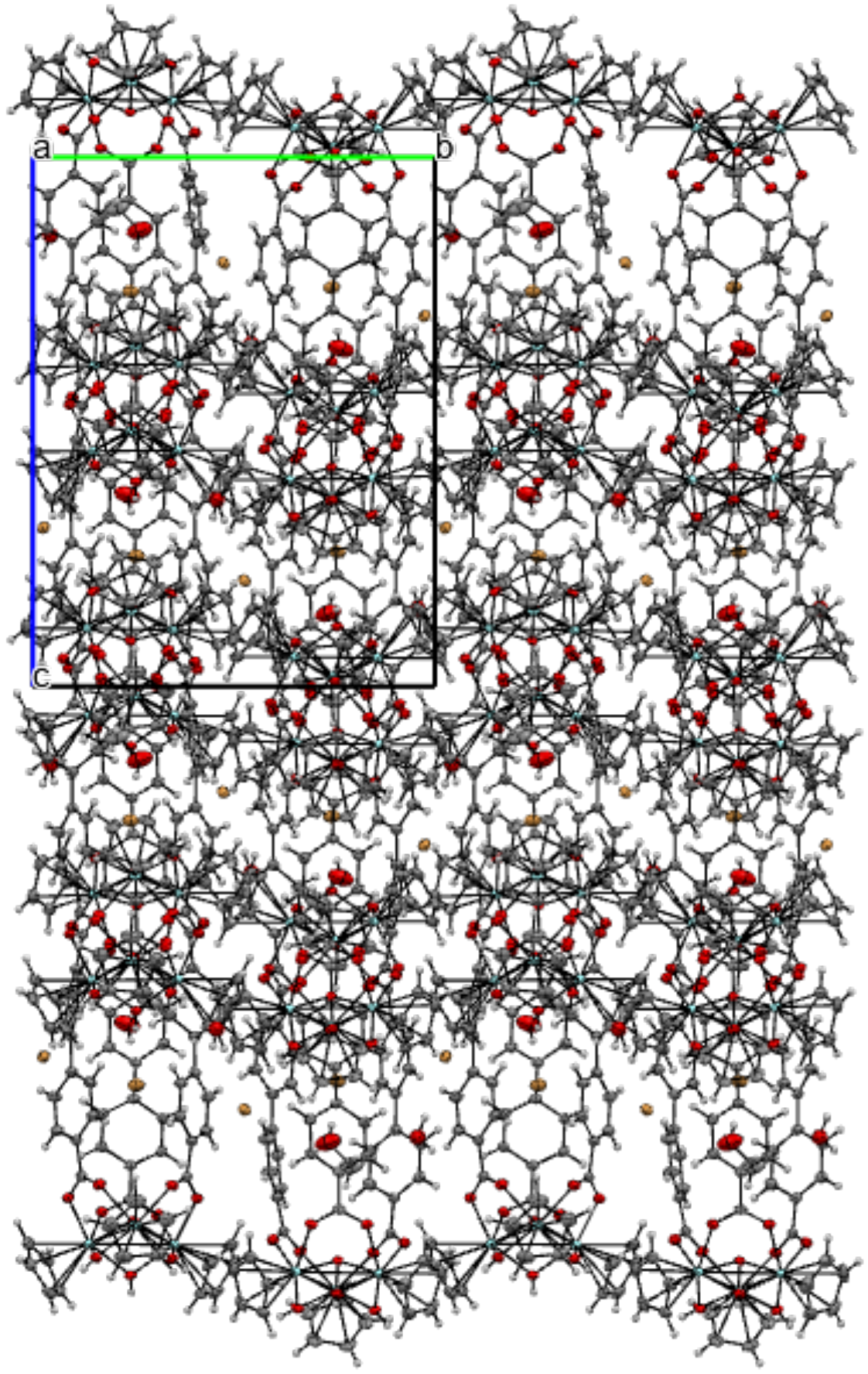

Figure S29. A packed unit cell of [cage][Br]$_2$-cig viewed along the crystallographic a-axis. The green and blue axes are the b- and c-axis respectively. All disordered parts are shown.

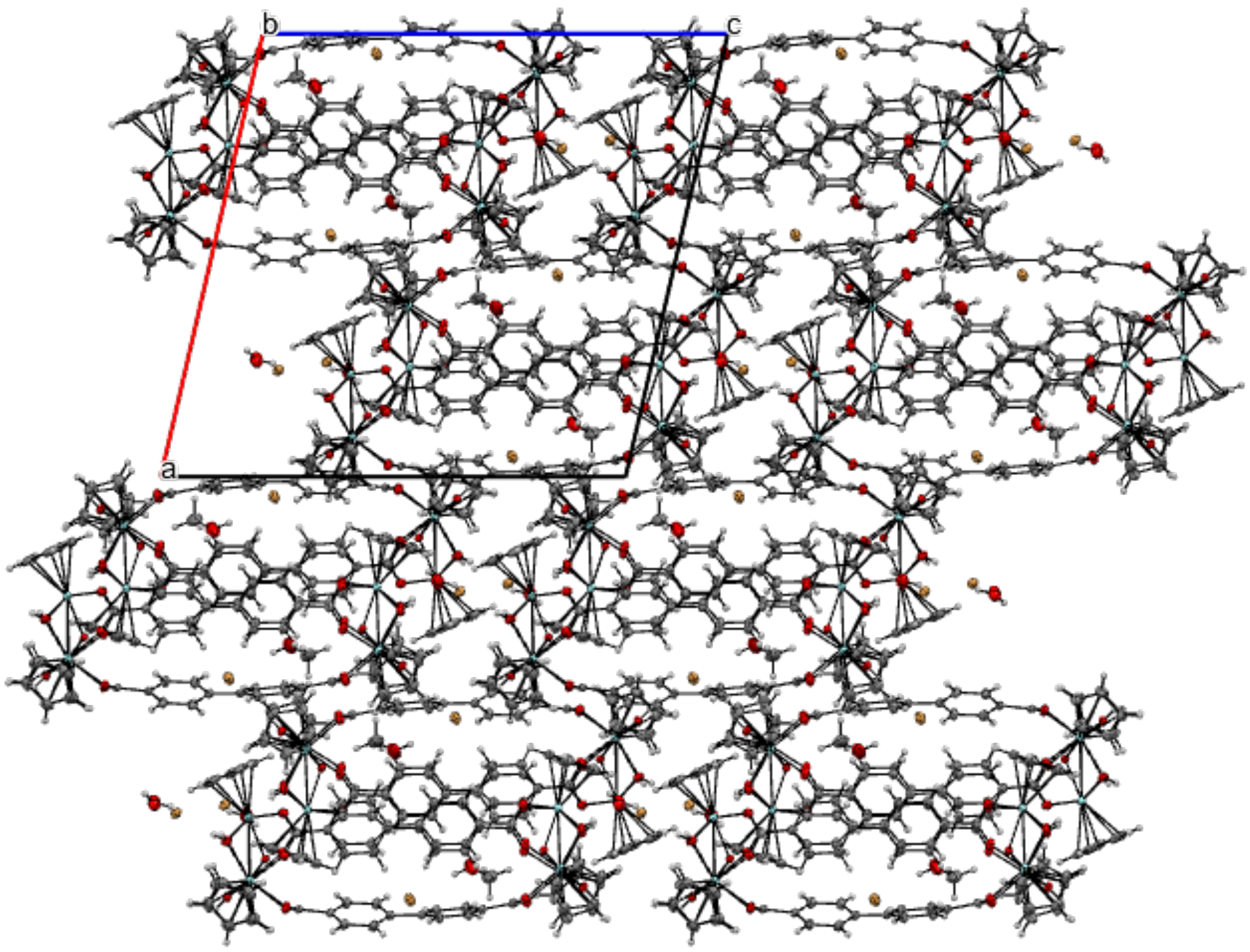

Figure S30. A packed unit cell of [cage][Br]$_2$-cig viewed along the crystallographic b-axis. The red and blue axes are the a- and c-axis respectively. All disordered parts are shown.

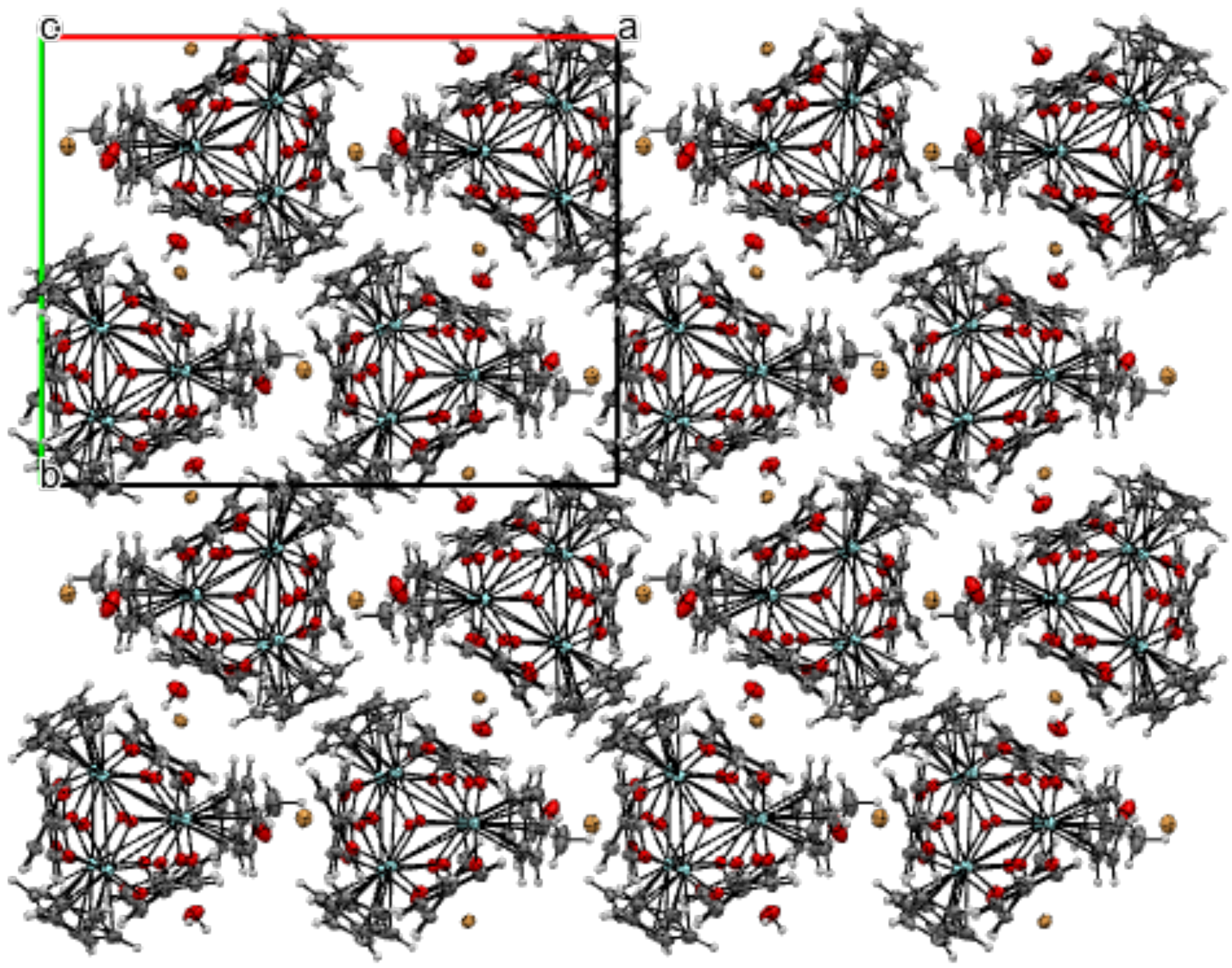

Figure S31. A packed unit cell of RS_26161 viewed along the crystallographic c-axis. The red and green axes are the a- and b-axis respectively. All disordered parts are shown.

Table S7. SCXRD crystal data and structure refinement for of [cage][Br]$_2$-cig.

Empirical formula	$C_{73}H_{66}Br_2O_{22}Zr_6$
Formula weight	2002.39
Crystal color, shape, size	Colorless block, 0.15 × 0.23 × 0.25 mm^3
Temperature	153(2) K
Crystal system, space group	Monoclinic, $P2_1/n$
Unit cell dimensions	a = 21.1656(7) Å α = 90°<br>b = 16.0251(6) Å β = 102.9950(10)°<br>c = 21.6374(7) Å γ = 90°
Volume	7151.0(4) Å^3
Z	4
Density (calculated)	1.86 g/cm^3
Absorption coefficient	2.037 mm^{-1}
F(000)	3960
Radiation	MoK_α (λ=0.71073 Å)
2θ range	3.98° to 61.11° (0.70 Å)
Index ranges	−30 ≤ h ≤ 30<br>−22 ≤ k ≤ 22<br>−30 ≤ l ≤ 30
Reflections collected	178490
Independent reflections	21890<br>R_{int} = 0.0686<br>R_{sigma} = 0.0408
Completeness to θ = 25.242°	99.9
Data / Restraints / Parameters	21890 / 1559 / 961
Absorption correction Tmin/Tmax	0.641 / 0.750 (Multi-Scan method)
Goodness-of-fit on F^2	1.035
Final R indexes [$I \geq 2\sigma(I)$]	R_1 = 0.0357<br>wR_2 = 0.0837
Final R indexes [all data]	R_1 = 0.0571<br>wR_2 = 0.0931
Largest peak/hole	1.05/−1.06 eÅ^{-3}

G. References

(1) B. Le Ouay, M. Tokiwa, R. Ohtani and M. Ohba, *Chem. Mater.*, 2025, **37**, 3414–3422.

(2) Bruker, *SAINT*, *V8.41*, Bruker AXS Inc., Madison, Wisconsin, USA.

(3) L. Krause, R. Herbst-Irmer, G. M. Sheldrick, D. Stalke, *J. Appl. Cryst.*, 2015, **48**, 3–10.

(4) G. M. Sheldrick, *Acta Cryst.*, 2015, **A71**, 3–8.

(5) G. M. Sheldrick, *Acta Cryst.*, 2015, **C71**, 3–8.

(6) A. L. Spek, *Acta Cryst.*, 2015, **C71**, 9–18.
